# Peripheral CD4^+^ T cells correlate with response and survival in patients with advanced non-small cell lung cancer receiving chemo-immunotherapy

**DOI:** 10.3389/fimmu.2024.1364507

**Published:** 2024-04-08

**Authors:** Xin Yang, Qiao Li, Tianyang Zeng

**Affiliations:** ^1^Department of Cardio-Thoracic Surgery, Deyang People’s Hospital, Deyang, Sichuan, China; ^2^Department of Pathology, Deyang People’s Hospital, Deyang, Sichuan, China; ^3^Department of Thoracic Surgery, The First Affiliated Hospital of Chongqing Medical University, Chongqing, China

**Keywords:** chemoimmunotherapy, lymphocyte subsets, biomarker, prognosis, non-small cell lung cancer

## Abstract

**Background:**

The aim of the present study was to explore the potential of peripheral immune cells in predicting the response and prognosis of patients with advanced non-small cell lung cancer (NSCLC) receiving anti-PD-1 immunotherapy and platinum-based chemotherapy.

**Participants and Methods:**

We utilized flow cytometry to examine the levels and dynamics of blood immune cells in 79 advanced NSCLC patients treated with the chemoimmunotherapy between December 2019 and January 2022. The pre- and post-treatment blood samples were collected within 3 days prior to the initiation of the first and third cycle of combination treatment, respectively. Progression-free survival (PFS) and overall survival (OS) analyses were conducted using Kaplan-Meier method and Cox regression models.

**Results:**

The pre-treatment CD4^+^/Total T cells ratio was significantly higher in responders than non-responders (*P <* 0.05). The levels of pre-treatment total lymphocytes (*P =* 0.012), total B lymphocytes (*P =* 0.025), and NK cells (*P =* 0.022), and post-treatment NK cells (*P =* 0.011) and NKT cells (*P =* 0.035) were significantly associated with OS. Post-treatment CD8^+^/Total T cells ratio was positively correlated with OS (*P =* 0.038). In multivariate analysis, post-treatment NK cells and post-treatment CD4^+^CD8^+^/Total T cells ratio were negatively associated with OS (hazard ratio [HR] = 10.30, *P =* 0.038) and PFS (HR = 1.95, *P =* 0.022), respectively. Notably, significantly positive correlations were observed between CD4^+^/Total T cells ratio and prognosis both before and after treatment (*P <* 0.05).

**Conclusion:**

To summarize, our finding reveals that high CD4^+^/total T cells ratio was associated with favorable response and prognosis, highlighting its potential as a predictive biomarker to guide the selection of likely responders to platinum and anti-PD-1 combination therapy.

## Introduction

Non-small cell lung cancer (NSCLC) is among the leading cause of cancer-related deaths worldwide, greatly endangering public health (1). Cytotoxic therapies, such as platinum-based chemotherapy, in combination with immune checkpoint inhibitors (ICIs) targeting PD-1/PD-L1 axis have been shown to profoundly improve efficacies of NSCLC treatment by synergizing to enhance anti-cancer immunity (2–4). Of note, only a limited range of NSCLC patients could derive significant survival benefits from the combination therapy (5, 6). However, specific biomarkers that were capable of predicting responses to the chemoimmunotherapy (chemoIO) remain to be identified (7–9). Therefore, it is paramount to identify feasible biomarkers to discriminate responders to the chemoIO from non-responders (10).

Peripheral blood might contain immune cells that were derived from the sites of tumor tissues, and therefore might have been recently recognized to possess predictive values for tumor infiltration and therapy response across multiple cancers, such as NSCLC, colorectal adenocarcinoma, endometrial adenocarcinoma and renal clear cell carcinoma (11). For example, increased lymphocyte-to-monocyte ratio in the peripheral blood was associated with improved clinical outcome in patients with metastatic nasopharyngeal carcinoma (12). High circulating NK cell counts forecasted a better overall survival in patients with untreated advanced gastric cancer (13). In addition, enhanced proliferation of peripheral PD-1^+^CD8^+^ T cells was linked with improved prognosis (14), while relative B cell levels in the blood predicted a poor overall survival in patients with NSCLC receiving immune checkpoint inhibitors-based therapy (15). However, the association between peripheral immune cells and clinical outcome remains to be explored in NSCLC patients treated with chemoIO.

Here, we present the first research centering on evaluating the relationship of the compositions of peripheral immune cells with the response and prognosis in patients with inoperable advanced NSCLC receiving chemoIO, with the aim of characterizing potential response biomarkers to chemoIO. Overall, our findings may pave the way for further research on identifying novel biomarkers in the peripheral blood to promote the implementation of chemo-immunotherapy in clinical practice for treating NSCLC.

## Patients and methods

### Study population

A total of 79 advanced NSCLC patients (stage III and IV at diagnosis according to IASLC 8th version) receiving platinum-based chemotherapy in combination with PD-1 checkpoint inhibitors at the First Affiliated Hospital of Chongqing Medical University (Chongqing, China) between September 2019 and January 2022 were enrolled in this study. Survival follow-up was carried out through multiple ways, mainly including clinic visits, reaching patients through monthly phone calls, and surveying death reports. Overall survival (OS) and Progression-free survival (PFS) were used as the endpoints for survival outcomes. OS was defined as the duration time starting from the initiation of chemoIO treatment to death from any cause. PFS was defined as the duration time starting from the initiation of chemoIO treatment to disease progression or death from any cause, whichever happened first. The follow-up period of OS and PFS ended December 11, 2022, or death.

### Criteria of inclusion and exclusion

The inclusion of patients was based on the following criteria: 1) stage III-IV NSCLC patients with diagnostic biopsy; 2) patients treated with platinum-based chemotherapy in combination with immune checkpoint inhibitors targeting PD-1; 3) patients without autoimmune diseases; 4) patients with normal functions of liver and kidney; 5) patients with good tolerance to chemotherapy plus immunotherapy, as indicated by Karnofsky performance status (KPS) score ≥ 80.

The exclusion criteria were as follows: 1) patients receiving adjuvant therapy after undergoing radical surgery of lung cancer; 2) patients treated with neoadjuvant therapy; 3) patients with severe organ dysfunctions; 3) patients with incomplete clinical data (for example, only tested for blood immune cells once); 4) patients with recent use of immunosuppressive medications; 5) patients lost to follow up; 6) positive for EGFR mutation, ALK fusion, and ROS1 fusion.

The following data were sourced from the medical records: age, gender, smoking history, histopathological features, disease stage, use of PD-1 inhibitors and comorbidity. Platinum-based chemotherapy was applied following the standard first-line chemotherapy regimen for advanced NSCLC. The immunotherapy drugs contained camrelizumab, tislelizumab, and pembrolizumab. Patients achieving complete response (CR) or partial response (PR) were grouped as responders, whereas patients showing stable disease (SD) or progressive disease (PD) were defined as non-responders, according to RECIST criteria v1.1.

### Patient sample collection

Fasting blood (> 200 μL) was aseptically collected through venipuncture within 3 days before the initiation of the first and third cycle of chemoIO treatment to examine pre- and post-treatment blood samples, respectively. Then, the blood was immediately transported in vacutainer blood collection tubes to the laboratory at room temperature and processed within 24 hours of draw.

### Flow cytometry analysis

**Figure 1:**
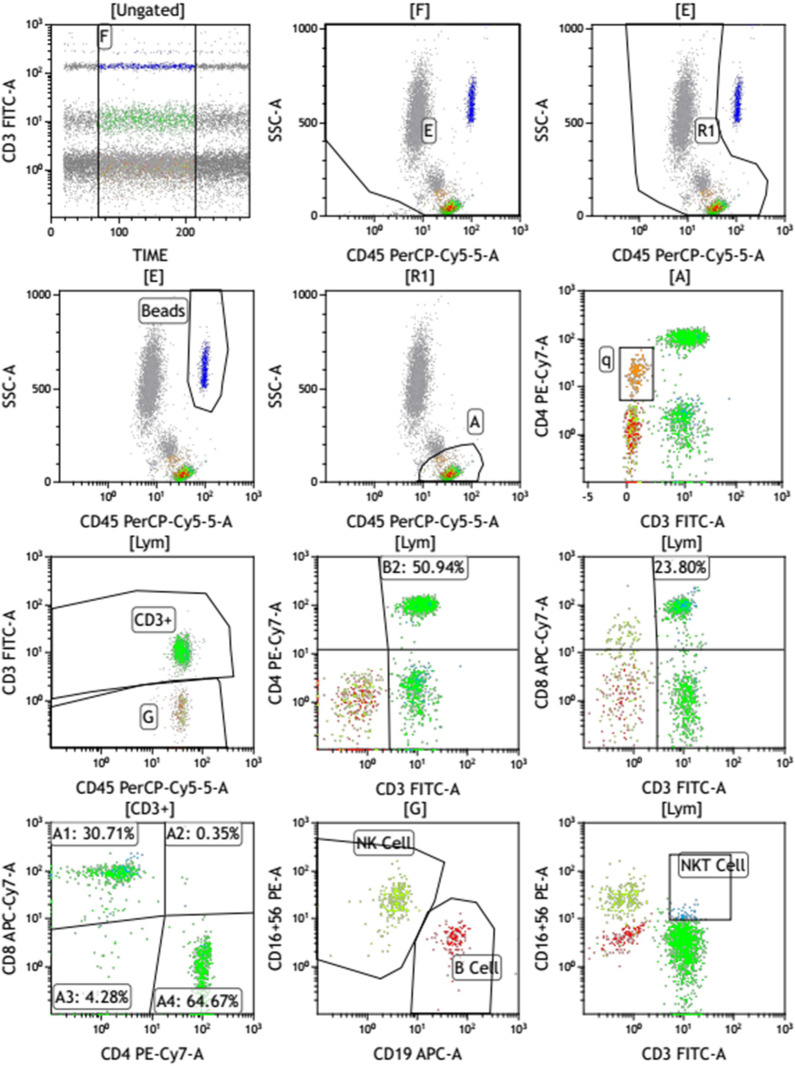
The gating strategy of a representative sample.

The flow cytometry was performed to determine the percentages and absolute counts of T lymphocytes (CD3^+^), B lymphocytes (CD19^+^), natural killer (NK) lymphocytes (CD3^–^CD16^+^ and/or CD56^+^), helper/inducer T lymphocytes (CD3^+^CD4^+^), and suppressor/cytotoxic T lymphocytes (CD3^+^CD8^+^) in the peripheral whole blood samples, using a 6-Color TBNK Reagent (QuantoBio, China, Z8610002) following the manufacturer’s instructions. Whole blood samples were stained within 24 hours of draw. The representative gating strategy for these cells from one representative patient was shown in **Figure 1**. Briefly, 50 μL of well-mixed, anticoagulated whole blood and 20 μL of CD3/CD16 + 56/CD45/CD4/CD19/CD8 reagent containing a mixture of fluorophore conjugated antibodies (fluorescein isothiocyanate (FITC)-labeled CD3 (UCHT1), phycoerythrin (PE)-labeled CD16 (CB16), PE-labeled CD56 (MEM-188), PerCP-Cy5.5–labeled CD45 (2D1), PC7–labeled CD4 (RPA-T4), allophycocyanin (APC)-labeled CD19 (HIB19), and APC-Cy7–labeled CD8 (HIT8a)) were pipetted into the bottom of the collection tubes, and vortexed gently to mix, followed by incubation in the dark at room temperature for 15–30 minutes. Next, to lyse red blood cells, 450 μL lysing solution were added to the tubes and incubated for 15–30 minutes in the dark at room temperature. Then, the samples were acquired on the flow cytometer (BD FACSCantoTMII, USA) and the data were analyzed using the BD FACSCantoTM clinical software.

To simply introduce the gating strategies, nucleated cells (R1) were first revealed by CD45 expression and side scatter (SSC) size. Then, the sum of lymphocytes and monocytes (A) were gated by CD45^high^ and SSC^low^ populations. Within the gate (A), the total lymphocytes (Lym) were identified by gating out monocytes in (q). The lymphocytes (Lym) could be split into CD3 positive T cells and CD3 negative cells (G) by the CD3 expression. CD3 positive T cells were then further identified and gated by the expression of CD4 and CD8 to identify helper and cytotoxic cells. Within the gate (G), CD3 negative cells were split into B cells and NK cells by the expression of CD19 and CD16 + 56. All information on antibodies was presented in **Supplementary Table S1**.

### Statistical analyses

GraphPad Prism version 10.0 and R software 3.6.2 were used for statistical analysis. Data were presented as mean ± SEM. Fisher’s exact test was used to analyze the categorical variables that were processed as percentages and frequencies. Survival analyses were performed using the Kaplan-Meier method with log-rank test and Cox proportional hazards regression model. The optimal cutpoints of different lymphocyte subsets for survival analysis were determined using the maximally selected test statistics from survminer R package. PFS used the same cutpoints as those of OS for corresponding immune cell subset. Hazard ratio (HR) and 95% confidence interval (CI) were calculated for Cox regression analysis. The variables that showed statistical significance in the univariate analyses were selected to be further analyzed in the multivariate models. The continuous variables were analyzed using Mann–Whitney U test and paired t-test, and the categorical variables using Fisher’s exact test. The two-sided probability of type I error was 0.05 in the analysis. *P <* 0.05 was determined to be with statistical difference.

## Results

### Patient demographic and clinical characteristics

**Figure 2:**
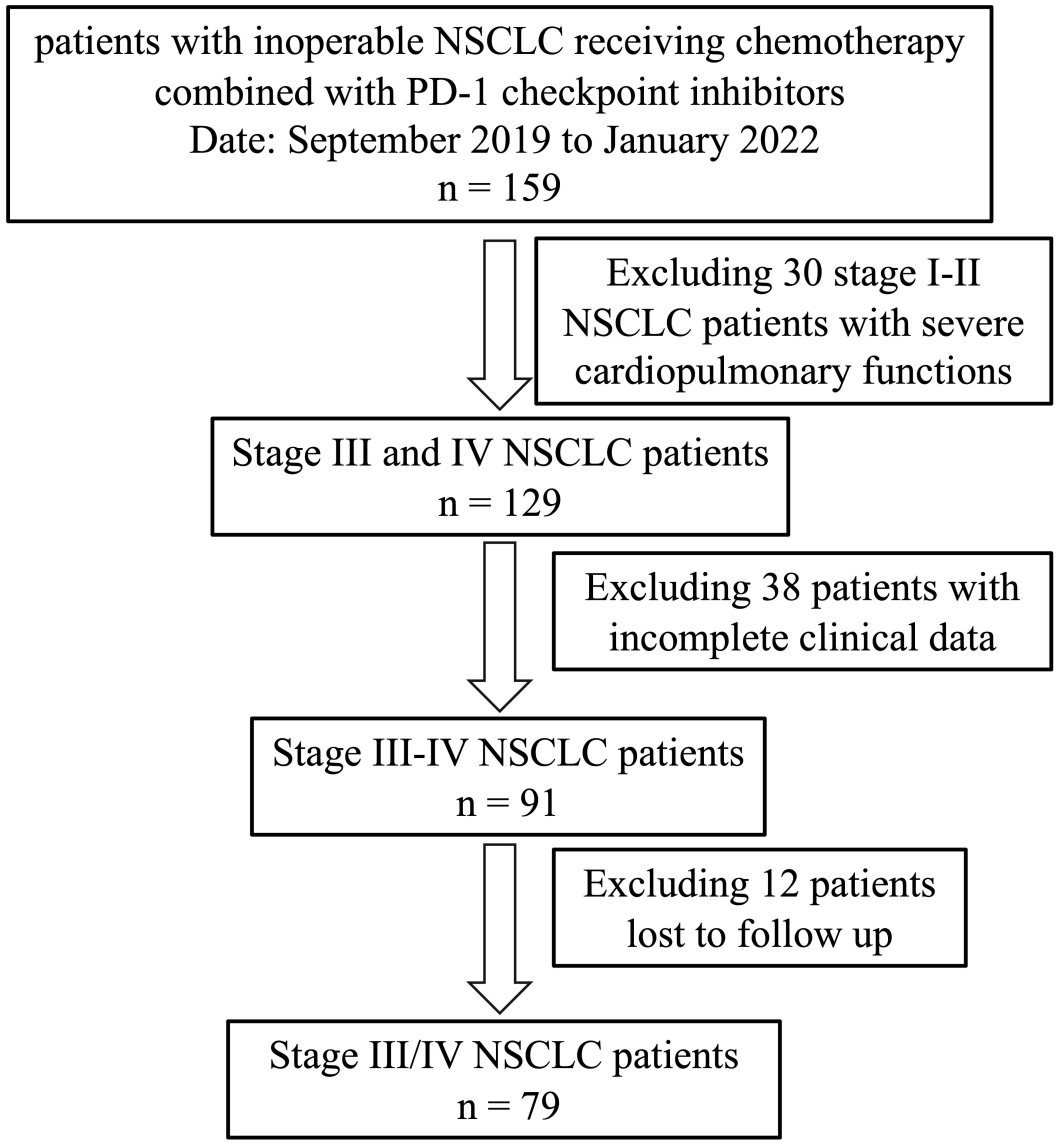
The flowchart of patient selection.

159 NSCLC patients who were not eligible for curative surgery and received combination treatment of platinum-based chemotherapy plus PD-1 checkpoint inhibitors were retrospectively enrolled from September 2019 to January 2022 at the Department of Thoracic Surgery, the First Affiliated Hospital of Chongqing Medical University. Thirty patients with stage I-II NSCLC were excluded due to severe cardiopulmonary functions. Next, 38 patients with incomplete clinical data were excluded. Twelve additional patients lost to follow up were excluded. Therefore, the study cohort comprised of 79 advanced NSCLC patients qualified for peripheral immune cell analysis (**Figure 2**). The detailed clinical characteristics of the included patients were displayed in **Table 1**. Across the 79 patients, 47 (59.5%) and 32 (40.5%) were lung squamous carcinoma and lung adenocarcinoma, respectively. The cohort has a median age of 59 years old (range, 30-74 years old), and 52 (65.8%) patients have a smoking history or currently smoke. Males, being a major part of the cohort, account for 89% of the cohort. According to the Fisher’s exact test, no significant differences of histology type, PD-1 inhibitors types, comorbidity, smoking history, age, and gender were observed between the two groups (CR/PR vs SD/PD) (**Table 1**). Overall, with 39 and 40 patients achieving CR/PR and SD/PD respectively, the objective response rate (ORR) was 49.4% (39/79) in advanced NSCLC patients treated with the chemoimmunotherapy.

**Table 1:** Baseline characteristics of 79 patients with advanced NSCLC.

Characteristic	Total (n =79)	CR/PR (n = 39)	SD/PD (n = 40)	*P*-value
Age				
< 59 years	38 (48%)	20	18	0.655
≥ 59 years	41 (52%)	19	22	
Gender				
Female	9 (11%)	4	5	1.000
Male	70 (89%)	35	35	
Smoking status				
History of smoking	52 (66%)	26	26	1.000
No history of smoking	27 (34%)	13	14	
Histopathological features				
Non-squamous	32 (41%)	15	17	0.820
Squamous cell carcinoma	47 (59%)	24	23	
Disease stage				
Stage III	48 (61%)	25	23	0.647
Stage IV	31 (39%)	14	17	
PD-1 inhibitors				
Camrelizumab	66 (83%)	32	34	0.876
Tislelizumab	11 (14%)	6	5	
Pembrolizumab	2 (3%)	1	1	
Comorbidity				
Hypertension	12 (15%)	7	5	0.688
Diabetes mellitus	10 (13%)	4	6	
Viral hepatitis B	5 (6%)	3	2	

p-values were estimated by Fisher’s exact test.

### Correlations between peripheral immune cells and response

**Figure 3:**
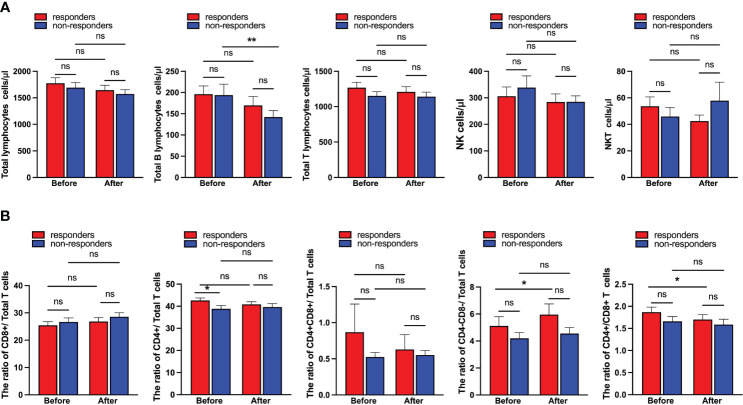
The correlations between pre- and post-treatment peripheral immune cell levels and responses. **(A)** The differneces of the abundances and **(B)** ratios of peripheral immune cell subsets between responders and non-responders. Responders group corresponds to complete response (CR) and partial response (PR), while non-responders group refers to stable disease (SD) and progressive disease (PD). The data were analyzed by paired t-test and Mann–Whitney U test. Data were presented as mean ± SEM. ^*^p<0.05 and ^**^p<0.01. ns, not significant.

The patients were stratified into two groups, responders (CR/PR) and non-responders (SD/PD) based on their response to platinum-based chemotherapy plus immunotherapy, as clarified by RECIST criteria v1.1. To investigate the association between clinical hematological parameters and patients’ response to therapy, the levels of peripheral total lymphocytes, total B lymphocytes, total T lymphocytes, NK cells and NKT cells were compared between CR/PR and SD/PD. The pre-treatment CD4^+^/Total T cells ratio (*P =* 0.003, Fisher’s exact test) and post-treatment CD4^-^CD8^-^ T cell frequency (*P =* 0.029, Fisher’s exact test) were significantly different between CR/PR group and SD/PD group (**Tables 2**, **3**). The level of peripheral total B lymphocytes was significantly decreased after treatment in non-responders as compared with pre-treatment (**Figure 3A**). Furthermore, the ratios of respective T cell subset to total or CD8^+^ T cells were analyzed. According to **Figure 3B**, pre-treatment CD4^+^/Total T cells ratio was significantly higher in responders than non-responders. In addition, CD4^-^CD8^-^/Total T cells ratio and the CD4^+^/CD8^+^ T cells ratio before treatment was significantly increased and decreased compared to their counterparts after treatment in responders, respectively (**Figure 3B**).

**Table 2:** Association of lymphocyte subsets before treatment with objective response rate.

Characteristics	Total (n =79)	CR/PR (n = 39)	SD/PD (n = 40)	*p*-value^a^
Total lymphocytes (cells/μL)	1714 (464, 3772)	1725 (464, 3772)	1561 (746, 3359)	0.551
> 1218		8	9	0.83
≤ 1218		31	31	
Total T lymphocytes (cells/μL)	1170 (399, 2854)	1278 (399, 2854)	1082.5 (480, 2241)	0.229
> 783		34	35	1
≤ 783		5	5	
B lymphocytes (cells/μL)	161 (27, 902)	179 (42, 653)	153 (27, 902)	0.943
> 128		26	26	0.876
≤ 128		13	14	
NK cells (cells/μL)	260 (21, 1376)	228 (21, 1017)	265.5 (60, 1376)	0.540
> 112		33	37	0.311
≤ 112		6	3	
NKT cells (cells/μL)	39 (6, 216)	44 (8, 216)	31 (6, 199)	0.432
> 69		8	9	0.83
≤ 69		31	31	
CD4^+^/CD8^+^ T cells ratio	1.6 (0.3, 3.53)	1.61 (0.93, 3.25)	1.585 (0.3, 3.53)	0.187
> 1.23		30	29	0.651
≤ 1.23		9	11	
CD4^+^/Total T cells ratio	41.77 (19.79, 60.7)	42.71 (30.25, 57.43)	38.61 (19.79, 60.7)	0.047
> 31.44		38	29	0.003
≤ 31.44		1	11	
CD8^+^/Total T cells ratio	25 (13.37, 65.15)	24.26 (13.37, 50.72)	25.355 (14.82, 65.15)	0.541
> 26.81		15	17	0.715
≤ 26.81		24	23	
CD4^+^CD8^+^/Total T cells ratio	0.44 (0, 15.51)	0.4 (0, 15.51)	0.445 (0.07, 1.72)	0.382
> 0.15		34	33	0.562
≤ 0.15		5	7	
CD4^-^CD8^-^/Total T cells ratio	3.91 (0.91, 23.99)	4.36 (0.91, 23.99)	3.575 (1.18, 15.46)	0.257
> 5.62		11	7	0.257
≤ 5.62		28	33	

a p-Values were estimated by Fisher’s exact test and Mann–Whitney U test for categorical variables and continuous variables, respectively.

**Table 3:** Association of lymphocyte subsets with objective response rates after treatment.

Characteristics	Total (n =79)	CR/PR (n = 39)	SD/PD (n = 40)	*p*-value^a^
Total lymphocytes (cells/μL)	1577 (311, 2954)	1608 (311, 2954)	1489.5 (879, 2896)	0.554
> 1885		15	10	0.198
≤ 1885		24	30	
Total T lymphocytes (cells/μL)	1127 (222, 2376)	1187 (222, 2376)	1105 (437, 2026)	0.508
> 1090		23	20	0.423
≤ 1090		16	20	
B lymphocytes (cells/μL)	127 (9, 749)	149 (9, 749)	116 (23, 450)	0.294
> 95		30	26	0.243
≤ 95		9	14	
NK cells (cells/μL)	249 (73, 928)	240 (78, 928)	264.5 (73, 672)	0.903
> 147		27	34	0.095
≤ 147		12	6	
NKT cells (cells/μL)	32 (5, 509)	33 (8, 112)	28.5 (5, 509)	0.306
> 21		28	22	0.122
≤ 21		11	18	
CD4^+^/CD8^+^ T cells ratio	1.57 (0.48, 3.7)	1.57 (0.61, 3.62)	1.475 (0.48, 3.7)	0.483
> 1.20		30	23	0.066
≤ 1.20		9	17	
CD4^+^/Total T cells ratio	39.58 (20.53, 60.92)	40.11 (26.65, 55.57)	39.555 (20.53, 60.92)	0.540
> 27.71		38	34	0.108
≤ 27.71		1	6	
CD8^+^/Total T cells ratio	26.28 (13.38, 51.91)	25.43 (13.38, 49.11)	27.65 (15, 51.91)	0.399
> 23.46		25	26	0.933
≤ 23.46		14	14	
CD4^+^CD8^+^/Total T cells ratio	0.4 (0.06, 8.02)	0.35 (0.06, 8.02)	0.415 (0.06, 1.83)	0.714
> 0.51		11	17	0.184
≤ 0.51		28	23	
CD4^-^CD8^-^/Total T cells ratio	4.05 (1.05,23.49)	4.67 (1.23,23.49)	3.915 (1.05,16.75)	0.123
> 5.00		19	10	0.029
≤ 5.00		20	30	

a p-Values were estimated by Fisher’s exact test Mann–Whitney U test for categorical variables and continuous variables, respectively.

### Correlations between peripheral immune cells and clinical outcomes

**Figure 4:**
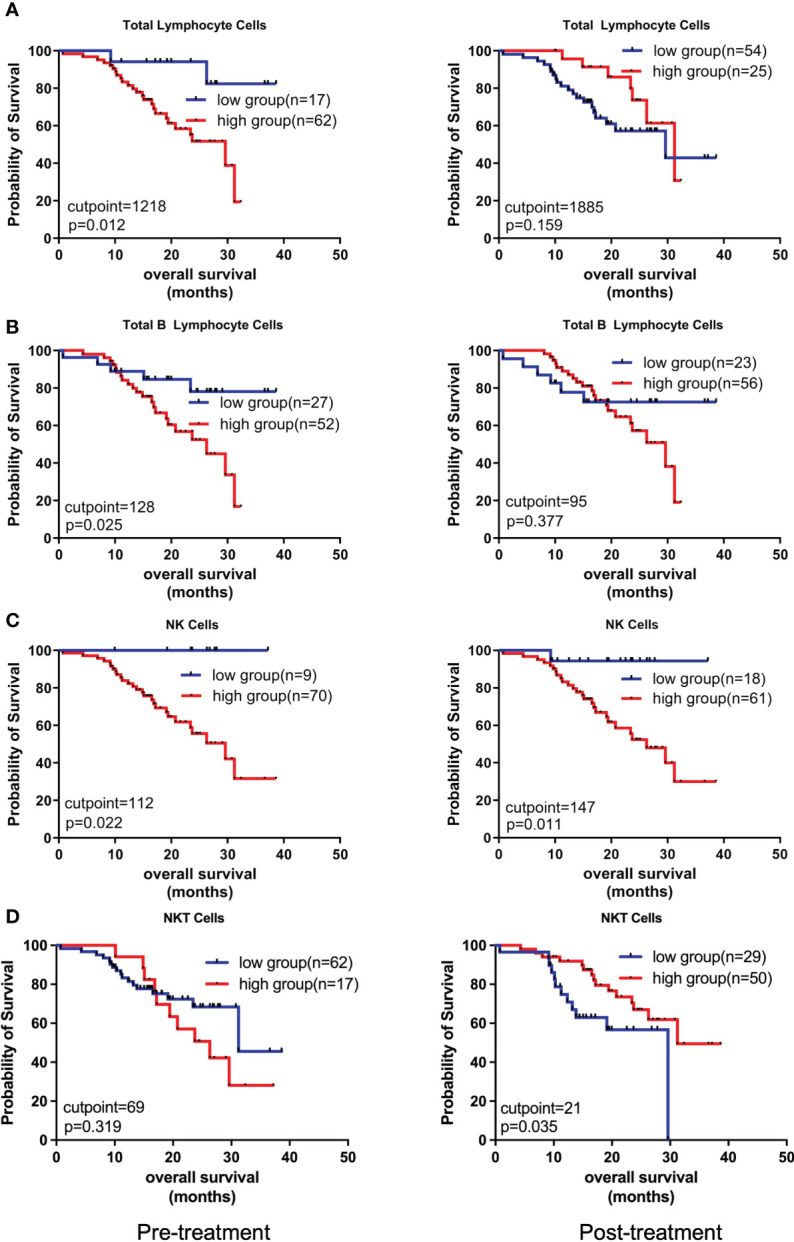
Kaplan-Meier analysis on the overall survival with regard to different peripheral immune cell subsets. The Kaplan-Meier curves of OS of patients stratified by total lymphocyte cells **(A)**, total B lymphocyte cells **(B)**, NK cells **(C)**, and NKT cells **(D)** at pre- and post-treatment according to the optimal cutpoints of respective lymphocyte subsets. The high and low groups were stratified by the cutpoints that were determined using the maximally selected test statistics for OS. The log-rank test was conducted to evaluate the significance of patients’ survival.

**Figure 5:**
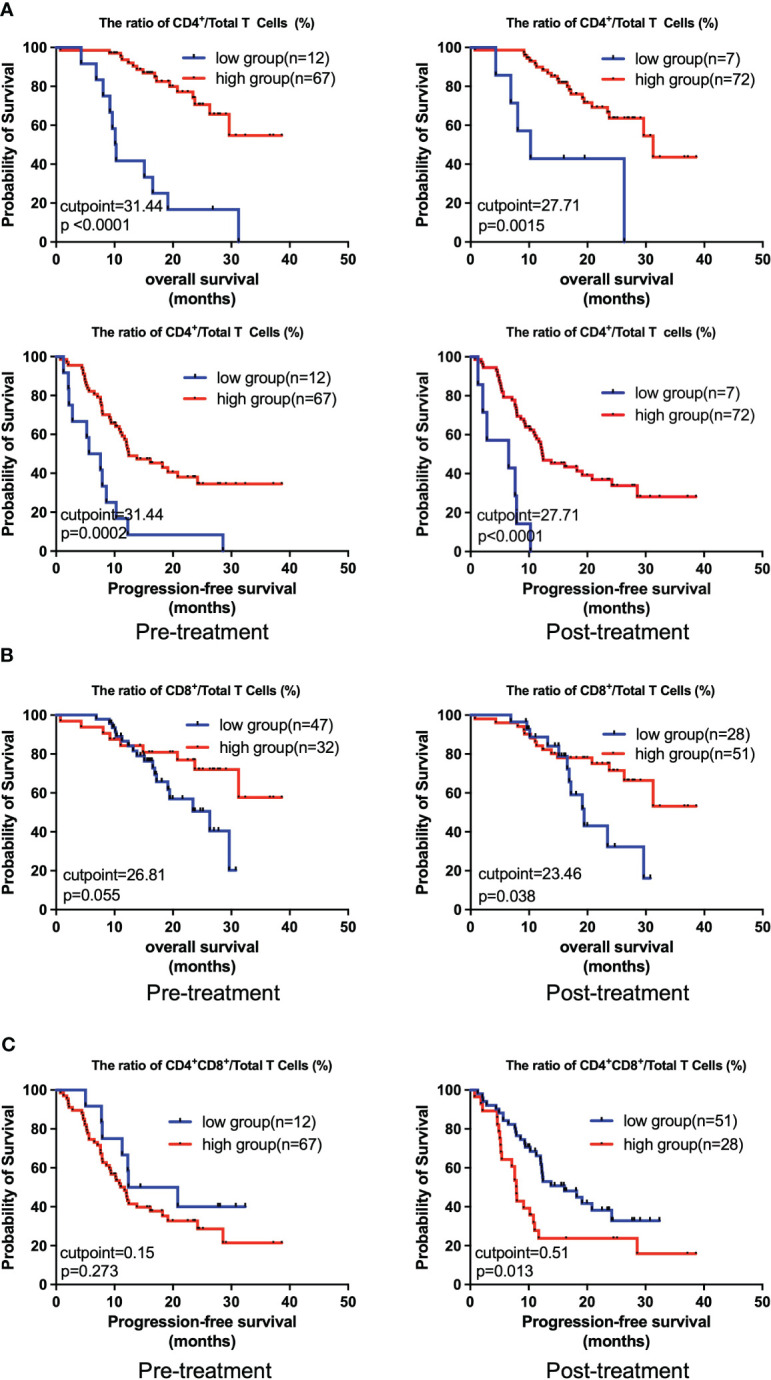
Kaplan-Meier analysis on the overall survival and progression-free survival with regard to the relative levels of different peripheral immune cells. The Kaplan-Meier curves of OS and PFS of patients stratified by CD4+/Total T cells ratio **(A)**, OS of patients stratified by CD4+/Total T cells ratio **(B)**, and PFS of patients stratified by CD4^+^CD8^+^/Total T cells ratio **(C)** at pre- and post-treatment according to the best cutpoints of respective immune cell ratios. The high and low groups in both OS and PFS analysis were stratified by the cutpoints that were determined using the maximally selected test statistics for OS. The log-rank test was conducted to evaluate the significance of patients’ survival.

Next, we comparatively analyzed the differences between the pre- and post-treatment peripheral immune cells compositions in NSCLC patients treated with chemoIO. Low pre-treatment levels of total lymphocytes (*P =* 0.012), total B lymphocytes (*P =* 0.025), and low pre- and post-treatment levels of NK cells (*P =* 0.0215; *P =* 0.011) were associated with significantly better overall survival than high-level groups, while higher levels of post-treatment NKT cells were correlated with longer overall survival (*P =* 0.035) (**Figure 4**, **Supplementary Figure S1**). However, no association was observed between peripheral immune cell levels and progression-free survival (**Supplementary Figure S2**). Next, the correlations between the relative abundances of immune cell subsets among the total T cells and patients’ prognosis were also analyzed. Both before and after chemoIO treatment, the CD4^+^/total T cells ratio was positively associated with improved OS (before: *P <* 0.001; after: *P =* 0.0015) and PFS (pre: *P =* 0.0002; post: *P <* 0.0001) (**Figure 5A**). And post-treatment CD8^+^/Total T cells ratio and CD4^+^CD8^+^/Total T cells ratio were significantly associated with OS and PFS, respectively (**Figures 5B, C**, **Supplementary Figures S3**, **S4**).

**Figure 6:**
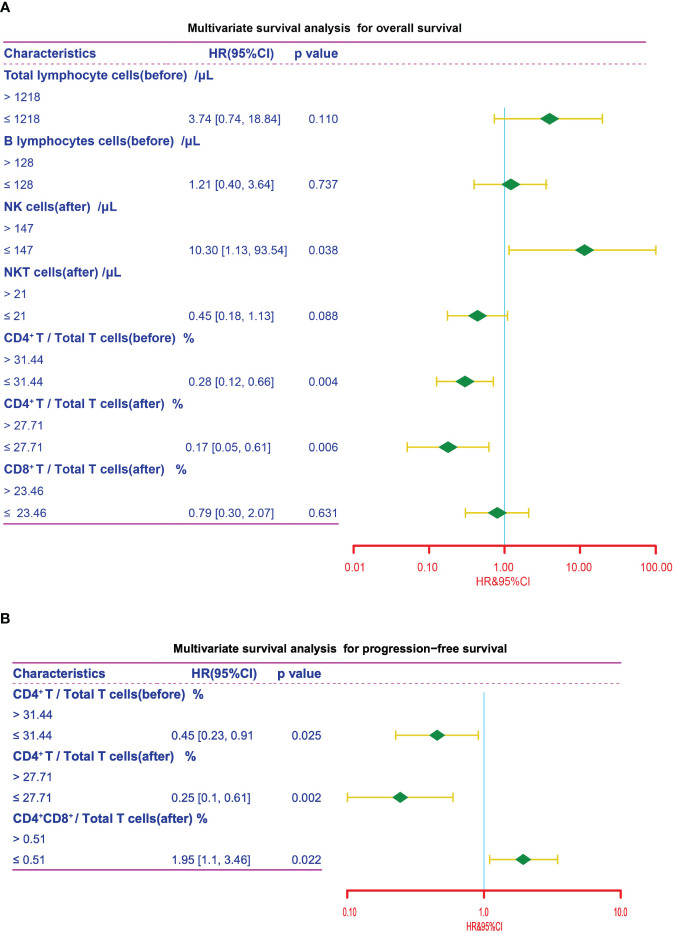
Multivariate survival analysis. Multivariate survival analysis of the peripheral immune cell subsets for OS **(A)** and PFS **(B)** in NSCLC patients for variables that showed statistical significance in univariate survival analysis. The cutpoints that were determined using the maximally selected test statistics.

We next conducted univariate and multivariate cox regression analysis to analyze the potential of peripheral lymphocyte subsets as independent prognostic factors. As illustrated by the forest plot, high level of post-treatment NK cells was significantly associated with shorter OS (*P =* 0.038, HR = 10.30). Conversely, pre-treatment CD4^+^/Total T cells ratio (*P =* 0.004, HR = 0.28) and post-treatment CD4^+^/Total T cells ratio (*P =* 0.006, HR = 0.17) were significantly associated with improved OS (**Figure 6A**, **Table 4**). Furthermore, an increased ratio of CD4^+^ to Total T cells before (*P =* 0.025, HR = 0.45) and after treatment (*P =* 0.002, HR = 0.25) was associated with favorable prognosis, while post-treatment CD4^+^CD8^+^/Total T cells ratio (*P =* 0.022, HR = 1.95) was independently associated with shorter PFS (**Figure 6B**, **Table 5**). Collectively, these results suggested that CD4^+^ T cells was associated with better clinical outcome in advanced NSCLC patients receiving the chemoIO treatment.

**Table 4:** Cox proportional analysis for overall survival.

Characteristics	Univariate			Multivariate		
	HR	95% CI	*p*-value	HR	95% CI	*p*-value
Total lymphocytes (before)	5.43	1.27 – 23.25	0.023	3.74	0.74 – 18.84	0.110
Total lymphocytes (after)	0.54	0.23 – 1.29	0.165			
Total T lymphocytes (before)	5.04	0.68 – 37.27	0.113			
Total T lymphocytes (after)	0.57	0.27 – 1.24	0.157			
Total B lymphocytes (before)	2.91	1.09 – 7.73	0.033	1.21	0.40 – 3.64	0.737
Total B lymphocytes (after)	1.51	0.60 – 3.78	0.381			
NK cells (before)	Inf	0 - Inf	0.998			
NK cells (after)	8.71	1.18 – 64.28	0.034	10.3	1.13 – 93.54	0.038
NKT cells (before)	1.49	0.68 – 3.28	0.322			
NKT cells (after)	0.44	0.20 – 0.96	0.04	0.45	0.18 – 1.13	0.088
CD4^+^/CD8^+^ T cells ratio (before)	1.77	0.69 – 4.53	0.236			
CD4^+^/CD8^+^ T cells ratio (after)	0.61	0.28 – 1.33	0.217			
CD4^+^/Total T cells (before)	0.16	0.07 – 0.035	0.000	0.28	0.12 – 0.66	0.004
CD4^+^/Total T cells (after)	0.23	0.09 – 0.62	0.004	0.17	0.05 – 0.61	0.006
CD8^+^/Total T cells (before)	0.44	0.19 – 1.04	0.061			
CD8^+^/Total T cells (after)	0.44	0.20 – 0.98	0.043	0.79	0.30 – 2.07	0.631
CD4^+^CD8^+^/Total T cells (before)	2.84	0.67 – 12.07	0.157			
CD4^+^CD8^+^/Total T cells (after)	1.68	0.79 – 3.59	0.181			
CD4^-^CD8^-^/Total T cells (before)	0.47	0.16 – 1.39	0.173			
CD4^-^CD8^-^/Total T cells (after)	0.51	0.22 – 1.22	0.13			

**Table 5:** Cox proportional analysis for progression-free survival.

Characteristics	Univariate			Multivariate		
	HR	95% CI	*p*-value	HR	95% CI	*p*-value
Total lymphocytes (before)	1.29	0.66 – 2.51	0.462			
Total lymphocytes (after)	0.57	0.30 – 1.09	0.089			
Total T lymphocytes (before)	1.07	0.48 - 2.38	0.87			
Total T lymphocytes (after)	0.58	0.33 – 1.02	0.057			
Total B lymphocytes (before)	1.09	0.61 - 1.94	0.768			
Total B lymphocytes (after)	0.99	0.54 - 1.81	0.981			
NK cells (before)	2.40	0.86 – 6.70	0.094			
NK cells (after)	1.51	0.76 - 3.02	0.244			
NKT cells (before)	0.93	0.49 – 1.79	0.838			
NKT cells (after)	0.57	0.32 – 1.01	0.054			
CD4^+^/CD8^+^ T cells ratio (before)	1.03	0.55 - 1.93	0.92			
CD4^+^/CD8^+^ T cells ratio (after)	0.64	0.36 – 1.13	0.121			
CD4^+^/Total T cells (before)	0.31	0.16 – 0.60	0.001	0.45	0.23 - 0.91	0.025
CD4^+^/Total T cells (after)	0.20	0.09 – 0.47	0.000	0.25	0.10 – 0.61	0.002
CD8^+^/Total T cells (before)	0.79	0.44 – 1.41	0.431			
CD8^+^/Total T cells (after)	0.84	0.47 – 1.50	0.557			
CD4^+^CD8^+^/Total T cells (before)	1.56	0.70 – 3.48	0.276			
CD4^+^CD8^+^/Total T cells (after)	2.01	1.15 – 3.53	0.015	1.95	1.10 – 3.46	0.022
CD4^-^CD8^-^/Total T cells (before)	0.68	0.34 – 1.36	0.272			
CD4^-^CD8^-^/Total T cells (after)	0.76	0.43 – 1.35	0.352			

## Discussion

Chemotherapy in combination with anti PD-1/PD-L1 antibodies has become a mainstay for patients with advanced non-small cell lung cancer (16). However, accurate selection of potential responders to the chemoIO remains challenging due to the wide variations in patients’ clinical responses to immunotherapy due to tumor heterogeneity. Here, we demonstrated that CD4^+^/total T cells ratio was significantly higher in CR/PR group than in SD/PD group. In addition, our study uncovered that the frequencies of circulating immune cells, including CD4^+^ T cells, CD8^+^ T cells and NK cells, were significantly associated with the overall survival and progression-free survival. Notably, this is the first study to support the characterization of CD4^+^ T cells as a potential prognostic parameter in inoperable advanced NSCLC patients receiving chemoimmunotherapy.

Combining chemotherapy with ICIs could enhance immunotherapy efficacy by exposing tumor neoantigens and priming immune cells, thus inducing immunogenic cell death (17–19). For example, chemotherapy could enhance cytotoxic T lymphocytes-mediated killing of cancer cells through immunogenic modulation (20). However, no available blood-based biomarkers associated with clinical outcome have been investigated as of now. Therefore, there is a pressing need to identify effective biomarkers to guide selection of NSCLC patients that might derive survival benefit from chemoIO treatment. Due to the invasive and time-consuming nature of histopathological evaluation which is currently a standard disease monitoring approach, identifying a novel blood-based biomarker that is non-invasive and easily accessible is of great clinical significance (21). In contrast with tumor tissues, the immune cells in the peripheral blood would provide a far more convenient sample source for patient selection and might offer a more comprehensive immune landscape of the whole tumor since they are circulated systemically (22, 23). Moreover, durable antitumor immune responses also require unrelenting immune cell recruitment from the peripheral blood (24, 25). However, the association between peripheral immune cell subsets and clinical outcomes in NSCLC patients receiving chemoimmunotherapy has remained elusive yet. A previous study showed that the levels of peripheral T cells and NK cells were closely related to the pathological response in 59 patients with resectable stage IIA-IIIB NSCLC treated with neoadjuvant chemoIO (26). Here, we demonstrated that the peripheral CD4^+^/Total T cells ratio was significantly higher in responders (CR and PR) as compared to non-responders (SD and PD). Consistently, a previous study demonstrated that the activated CD4^+^ T cell subset in the peripheral blood was a potent mediator of anti-tumor immunity (27). Collectively, the present study demonstrated the association between peripheral CD4^+^ T cells and response to chemoIO in inoperable NSCLC patients for the first time.

The immune contexture is a major determinant of tumor progression and clinical outcomes in patients with solid tumors (28). For example, increased tumor-infiltrating lymphocytes (TILs) were associated with survival in patients with breast cancer (29). Besides, it has been reported that long-term responders showed significantly higher levels of peripheral CD62L^low^CD4^+^ T cells before PD-1 blockade therapy in patients with NSCLC (30). Moreover, the prognostic impact of the peripheral neutrophils-to-lymphocytes ratio has also been recognized across different cancers (31). Hematological biomarkers, which allow for longitudinal monitoring of real-time disease status by safe venipuncture, present an informative surrogate of histopathological examination for risk stratification and treatment guidance (32). For instance, as one of the most prevalent biomarkers used in liquid biopsy, ctDNA is now widely used to aid in the selection of NSCLC patients who might benefit from epidermal growth factor receptor (EGFR)-targeted therapy (33). Here, we demonstrated the association of lymphocytes and NK cells with prognosis in NSCLC patients receiving chemoimmunotherapy for the first time. Our study revealed that higher percentages of pre- and post-chemoIO CD4^+^ T cells were independently associated with improved OS and PFS in patients with NSCLC, which was in line with our finding that CD4^+^/Total T cells ratio before chemoIO therapy was higher in responders than non-responders. Taken together, these results suggested that peripheral CD4^+^ T cell subset might exert protective functions in response to chemoIO treatment, thus underscoring its potential as a predictive biomarker for screening beneficiaries before chemoIO treatment and evaluating efficacy after the chemoIO treatment. Despite less understood than CD8^+^ T cells in anti-cancer function, the CD4^+^ T cell subset has been recently demonstrated to be protective against cancer progression likely by enhancing tumoricidal activity of other antitumor effector cells subsets (34). For example, CD4^+^ T cell depletion retarded tumor growth by increasing effector T cell function (35). Moreover, it was lately demonstrated that a novel CD62L^low^CCR4^-^CCR6^+^ CD4^+^ T cell metacluster exhibited predictive potential of the immune status and sensitivity to PD-1 blockade (36). It should be noted though that effective prediction will most likely be satisfactorily achieved by comprehensively implementing multiple biomarkers instead of a single one, thus highlighting the importance of combining peripheral CD4+ T cells with other parameters to effectively evaluate efficacy and prognosis in response to chemoIO. Overall, this is the first study to suggest a positive correlation of peripheral CD4^+^ T cells with OS and PFS in patients with inoperable advanced NSCLC treated with chemoimmunotherapy.

However, there are several limits to the present study. Firstly, a prospective study should be conducted in the future to validate the relationships of the peripheral blood immune cell subsets to the response and prognostic outcomes in a larger cohort of NSCLC patients receiving chemoIO. Secondly, the functions of each immune cell subset are multifaceted, therefore a more detailed landscape of the immune composition need to be further profiled to investigate the specific immune subpopulation that is involved in cancer-related immunity. Lastly, with only relevance analysis being performed in the current study, experiments *in vitro* and *in vivo* will also be our next step to explore the molecular mechanisms underlying the protective roles of CD4^+^ T cells in patients with advanced NSCLC receiving chemo-immunotherapy.

In conclusion, with the prospects for long-time survival greatly improved by immunotherapy, our results provide timely and valuable information on the prognostic roles of CD4^+^ T cells in advanced NSCLC patients treated with chemoIO. Dynamic and longitudinal monitoring of the peripheral CD4^+^ T cells might aid in selection of likely responders to the treatment. A prospective study in a larger cohort of advanced NSCLC patients treated with chemoimmunotherapy is further warranted to validate the use of peripheral CD4^+^ T cells as biomarkers that are truly predictive of prognosis.

## Data availability statement

The original contributions presented in the study are included in the article/**Supplementary Material**. Further inquiries can be directed to the corresponding author.

## Ethics statement

The studies involving humans were approved by the Ethics Committee in the First Affiliated Hospital of Chongqing Medical University. The studies were conducted in accordance with the local legislation and institutional requirements. The participants provided their written informed consent to participate in this study.

## Author contributions

QL: Funding acquisition, Investigation, Writing – original draft, Data curation, Formal analysis, Writing – review & editing. XY: Conceptualization, Data curation, Formal analysis, Investigation, Methodology, Software, Visualization, Writing – review & editing, Writing – original draft. TZ: Conceptualization, Funding acquisition, Supervision, Writing – review & editing.

## Supplementary material

Supplementary Figure S1 Kaplan-Meier analysis on the overall survival. The Kaplan-Meier curves of OS of patients stratified by the optimal cutpoint of total T lymphocyte cells at pre- and post-treatment.

Supplementary Figure S2 Kaplan-Meier analysis on the progression-free survival. The Kaplan-Meier curves of PFS of patients stratified by the optimal cutpoints of OS at pre- and post-treatment.

Supplementary Figure S3 Kaplan-Meier analysis on the overall survival. The Kaplan-Meier curves of OS of patients stratified by the optimal cutpoints of OS at pre- and post-treatment.

Supplementary Figure S4 Kaplan-Meier analysis on the progression-free survival. The Kaplan-Meier curves of PFS at pre- and post-treatment. The cutpoints for PFS were determined by the optimal cutpoints of OS.

## References

[1] Siegel R Miller K Wagle N Jemal A. Cancer statistics 2023. CA: Cancer J Clin. (2023) 73:17–48. doi: 10.3322/caac.21763 36633525

[2] Kanda S Goto K Shiraishi H Kubo E Tanaka A Utsumi H. Safety and efficacy of nivolumab and standard chemotherapy drug combination in patients with advanced non-small-cell lung cancer: a four arms phase Ib study. Ann Oncol. (2016) 27:2242–50. doi: 10.1093/annonc/mdw416 PMC5178141 27765756

[3] Mathew M Enzler T Shu C Rizvi N. Combining chemotherapy with PD-1 blockade in NSCLC. Pharmacol Ther. (2018) 186:130–7. doi: 10.1016/j.pharmthera.2018.01.003 29352857

[4] Ribas A Wolchok J. Cancer immunotherapy using checkpoint blockade. Sci (New York N.Y.). (2018) 359:1350–5. doi: 10.1126/science.aar4060 PMC7391259 29567705

[5] Ren D Hua Y Yu B Ye X He Z Li C. Predictive biomarkers and mechanisms underlying resistance to PD1/PD-L1 blockade cancer immunotherapy. Mol Cancer. (2020) 19:19. doi: 10.1186/s12943-020-1144-6 32000802 PMC6993488

[6] Passaro A Brahmer J Antonia S Mok T Peters S. Managing resistance to immune checkpoint inhibitors in lung cancer: treatment and novel strategies. J Clin Oncol. (2022) 40:598–610. doi: 10.1200/JCO.21.01845 34985992

[7] Ma W Gilligan B Yuan J Li T. Current status and perspectives in translational biomarker research for PD-1/PD-L1 immune checkpoint blockade therapy. J Hematol Oncol. (2016) 9:47. doi: 10.1186/s13045-016-0277-y 27234522 PMC4884396

[8] Topalian S Taube J Pardoll D. Neoadjuvant checkpoint blockade for cancer immunotherapy. Sci (New York N.Y.). (2020) 367:6477. doi: 10.1126/science.aax0182 PMC7789854 32001626

[9] Rolfo C Russo A. In search of lost biomarker for immunotherapy in small-cell lung cancer. Clin Cancer Res. (2023) 30:652-4. doi: 10.1158/1078-0432.CCR-23-3087 38085269

[10] Somasundaram A Burns T. The next generation of immunotherapy: keeping lung cancer in check. J Hematol Oncol. (2017) 10:87. doi: 10.1186/s13045-017-0456-5 28434399 PMC5402056

[11] Wu T Madireddi S De Almeida P Banchereau R Chen Y Chitre A. Peripheral T cell expansion predicts tumour infiltration and clinical response. Nature. (2020) 579:274–8. doi: 10.1038/s41586-020-2056-8 32103181

[12] Jiang R Cai X Yang Z Yan Y Zou X Guo L. Elevated peripheral blood lymphocyte-to-monocyte ratio predicts a favorable prognosis in the patients with metastatic nasopharyngeal carcinoma. Chin J Cancer. (2015) 34:237–46. doi: 10.1186/s40880-015-0025-7 PMC4593366 26067059

[13] Pernot S Terme M Radosevic-Robin N Castan F Badoual C Marcheteau E. Infiltrating and peripheral immune cell analysis in advanced gastric cancer according to the Lauren classification and its prognostic significance. Gastric Cancer. (2020) 23:73–81. doi: 10.1007/s10120-019-00983-3 31267360

[14] Kamphorst A Pillai R Yang S Nasti T Akondy R Wieland A. Proliferation of PD-1+ CD8 T cells in peripheral blood after PD-1-targeted therapy in lung cancer patients. Proc Natl Acad Sci United States America. (2017) 114:4993–8. doi: 10.1073/pnas.1705327114 PMC5441721 28446615

[15] Xu X Wang D Chen W Li N Suwinski R Rossi A. A nomogram model based on peripheral blood lymphocyte subsets to assess the prognosis of non-small cell lung cancer patients treated with immune checkpoint inhibitors. Trans Lung Cancer Res. (2021) 10:4511–25. doi: 10.21037/tlcr PMC8743511 35070757

[16] Lahiri A Maji A Potdar P Singh N Parikh P Bisht B. Lung cancer immunotherapy: progress, pitfalls, and promises. Mol Cancer. (2023) 22:40. doi: 10.1186/s12943-023-01740-y 36810079 PMC9942077

[17] West H Mccleod M Hussein M Morabito A Rittmeyer A Conter H. Atezolizumab in combination with carboplatin plus nab-paclitaxel chemotherapy compared with chemotherapy alone as first-line treatment for metastatic non-squamous non-small-cell lung cancer (IMpower130): a multicentre, randomised, open-label, phase 3 trial. Lancet Oncol. (2019) 20:924–37. doi: 10.1016/S1470-2045(19)30167-6 31122901

[18] Limagne E Nuttin L Thibaudin M Jacquin E Aucagne R Bon M. MEK inhibition overcomes chemoimmunotherapy resistance by inducing CXCL10 in cancer cells. Cancer Cell. (2022) 40:136–152.e12. doi: 10.1016/j.ccell.2021.12.009 35051357

[19] Chen G Li X Li R Wu K Lei Z Dai R. Chemotherapy-induced neoantigen nanovaccines enhance checkpoint blockade cancer immunotherapy. ACS nano. (2023) 17:18818–31. doi: 10.1021/acsnano.3c03274 37750443

[20] Hodge J Garnett C Farsaci B Palena C Tsang K Ferrone S. Chemotherapy-induced immunogenic modulation of tumor cells enhances killing by cytotoxic T lymphocytes and is distinct from immunogenic cell death. Int J Cancer. (2013) 133:624–36. doi: 10.1002/ijc.28070 PMC3663913 23364915

[21] Lehrich B Zhang J Monga S Dhanasekaran R. Battle of the Biopsies: Role of tissue and liquid biopsy in hepatocellular carcinoma. J hepatology. (2023) 80:515-30. doi: 10.1016/j.jhep.2023.11.030 PMC10923008 38104635

[22] Luo H Wei W Ye Z Zheng J Xu R. Liquid biopsy of methylation biomarkers in cell-free DNA. Trends Mol Med. (2021) 27:482–500. doi: 10.1016/j.molmed.2020.12.011 33500194

[23] Luoma A Suo S Wang Y Gunasti L Porter C Nabilsi N. Tissue-resident memory and circulating T cells are early responders to pre-surgical cancer immunotherapy. Cell. (2022) 185:2918–2935.e29. doi: 10.1016/j.cell.2022.06.018 35803260 PMC9508682

[24] Spitzer M Carmi Y Reticker-Flynn N Kwek S Madhireddy D Martins M. Systemic immunity is required for effective cancer immunotherapy. Cell. (2017) 168:487–502.e15. doi: 10.1016/j.cell.2016.12.022 28111070 PMC5312823

[25] Hiam-Galvez K Allen B Spitzer M. Systemic immunity in cancer. Nat Rev Cancer. (2021) 21:345–59. doi: 10.1038/s41568-021-00347-z PMC8034277 33837297

[26] Ma T Wen T Cheng X Wang Y Wei P Yang B. Pathological complete response to neoadjuvant chemoimmunotherapy correlates with peripheral blood immune cell subsets and metastatic status of mediastinal lymph nodes (N2 lymph nodes) in non-small cell lung cancer. Lung Cancer (Amsterdam Netherlands). (2022) 172:43–52. doi: 10.1016/j.lungcan.2022.08.002 35988509

[27] Speiser D Chijioke O Schaeuble K Münz C. CD4 T cells in cancer. Nat Cancer. (2023) 4:317–29. doi: 10.1038/s43018-023-00521-2 36894637

[28] Bruni D Angell H Galon J. The immune contexture and Immunoscore in cancer prognosis and therapeutic efficacy. Nat Rev Cancer. (2020) 20:662–80. doi: 10.1038/s41568-020-0285-7 32753728

[29] Denkert C Von Minckwitz G Darb-Esfahani S Lederer B Heppner B Weber K. Tumour-infiltrating lymphocytes and prognosis in different subtypes of breast cancer: a pooled analysis of 3771 patients treated with neoadjuvant therapy. Lancet Oncol. (2018) 19:40–50. doi: 10.1016/S1470-2045(17)30904-X 29233559

[30] Kagamu H Kitano S Yamaguchi O Yoshimura K Horimoto K Kitazawa M. CD4 T-cell immunity in the peripheral blood correlates with response to anti-PD-1 therapy. Cancer Immunol Res. (2020) 8:334–44. doi: 10.1158/2326-6066.CIR-19-0574 31871122

[31] Templeton A Mcnamara M Šeruga B Vera-Badillo F Aneja P Ocaña A. Prognostic role of neutrophil-to-lymphocyte ratio in solid tumors: a systematic review and meta-analysis. J Natl Cancer Institute. (2014) 106:dju124. doi: 10.1093/jnci/dju124 24875653

[32] Tivey A Church M Rothwell D Dive C Cook N. Circulating tumour DNA - looking beyond the blood. Nat Rev Clin Oncol. (2022) 19:600–12. doi: 10.1038/s41571-022-00660-y PMC9341152 35915225

[33] Donaldson J Park B. Circulating tumor DNA: measurement and clinical utility. Annu Rev Med. (2018) 69:223–34. doi: 10.1146/annurev-med-041316-085721 28846488

[34] Miggelbrink A Jackson J Lorrey S Srinivasan E Waibl-Polania J Wilkinson D. CD4 T-cell exhaustion: does it exist and what are its roles in cancer? Clin Cancer Res. (2021) 27:5742–52. doi: 10.1158/1078-0432.CCR-21-0206 PMC8563372 34127507

[35] Chen Y Li P Pan N Gao R Wen Z Zhang T. Tumor-released autophagosomes induces CD4 T cell-mediated immunosuppression via a TLR2-IL-6 cascade. J immunotherapy Cancer. (2019) 7:178. doi: 10.1186/s40425-019-0646-5 PMC6625067 31300052

[36] Kagamu H Yamasaki S Kitano S Yamaguchi O Mouri A Shiono A. Single-cell analysis reveals a CD4+ T-cell cluster that correlates with PD-1 blockade efficacy. Cancer Res. (2022) 82:4641–53. doi: 10.1158/0008-5472.CAN-22-0112 PMC9755963 36219677

