# Supplementary material for: Peripheral CD4+ T cells correlate with response and survival in patients with advanced non-small cell lung cancer receiving chemo-immunotherapy
Source: Front Immunol. 2024 Apr 8;15:1364507. doi: 10.3389/fimmu.2024.1364507 (PMC11033411; doi:10.3389/fimmu.2024.1364507)
Supplement: Supplementary file 1 [file DataSheet_1.pdf]

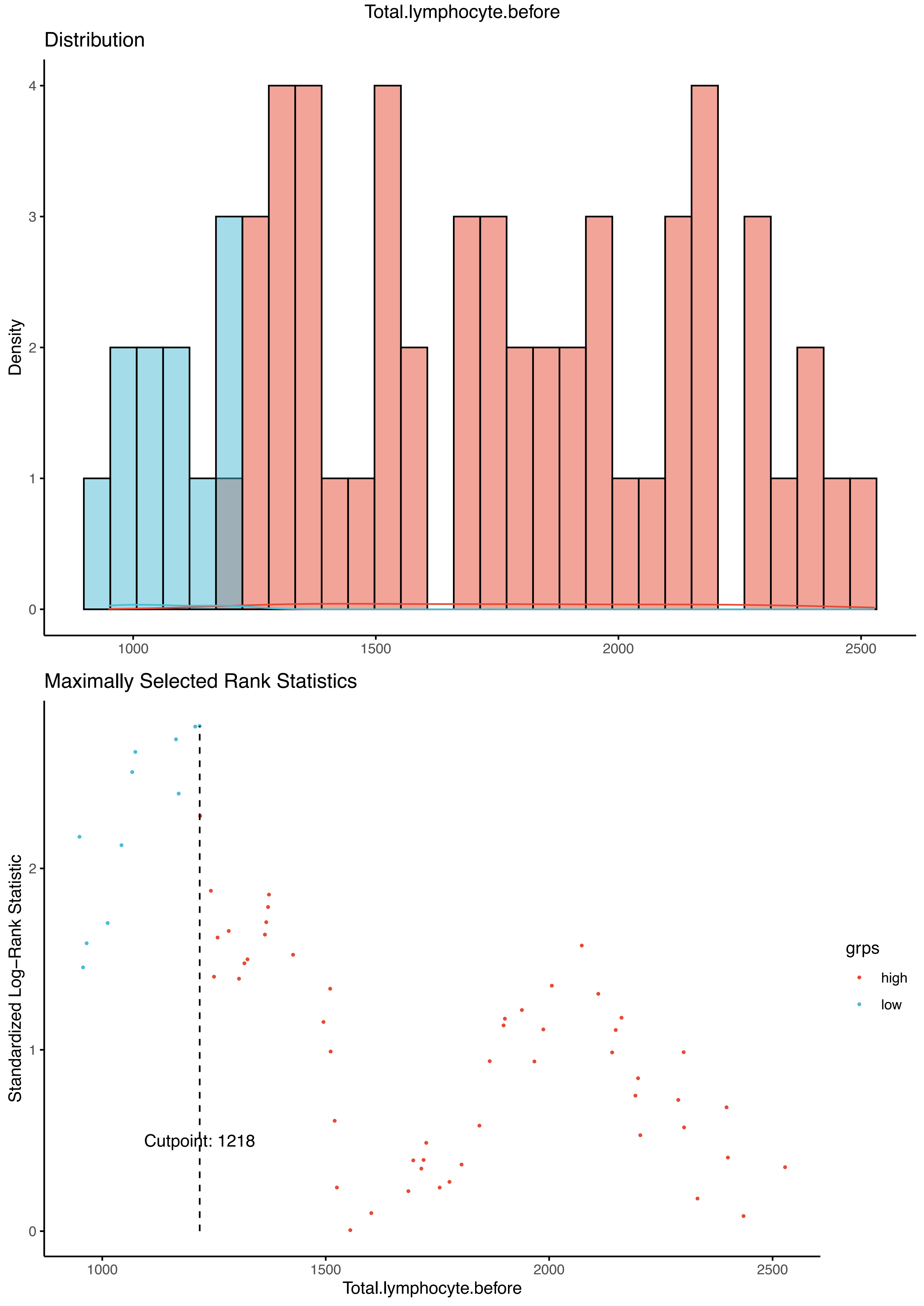

Total.lymphocyte.before
Distribution
Density
0
1
2
3
4
1000
1500
2000
2500
Maximally Selected Rank Statistics
Standardized Log−Rank Statistic
0
1
2
Cutpoint: 1218
1000
1500
2000
2500
Total.lymphocyte.before
grps
high
low

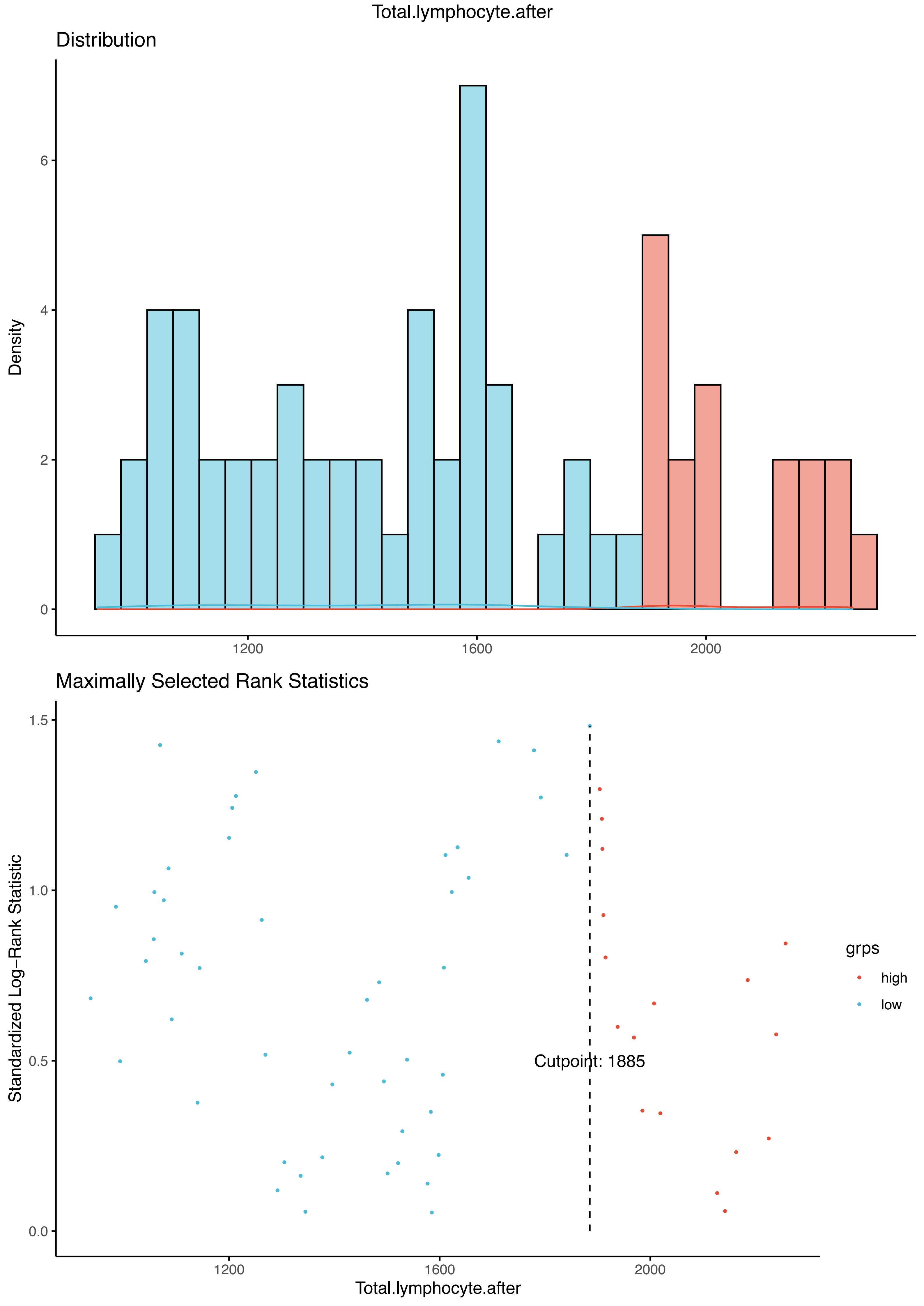

Total.lymphocyte.after
Distribution
Density
0
2
4
6
1200
1600
2000
Maximally Selected Rank Statistics
Standardized Log−Rank Statistic
0.0
0.5
1.0
1.5
Cutpoint: 1885
1200
1600
2000
Total.lymphocyte.after
grps
high
low

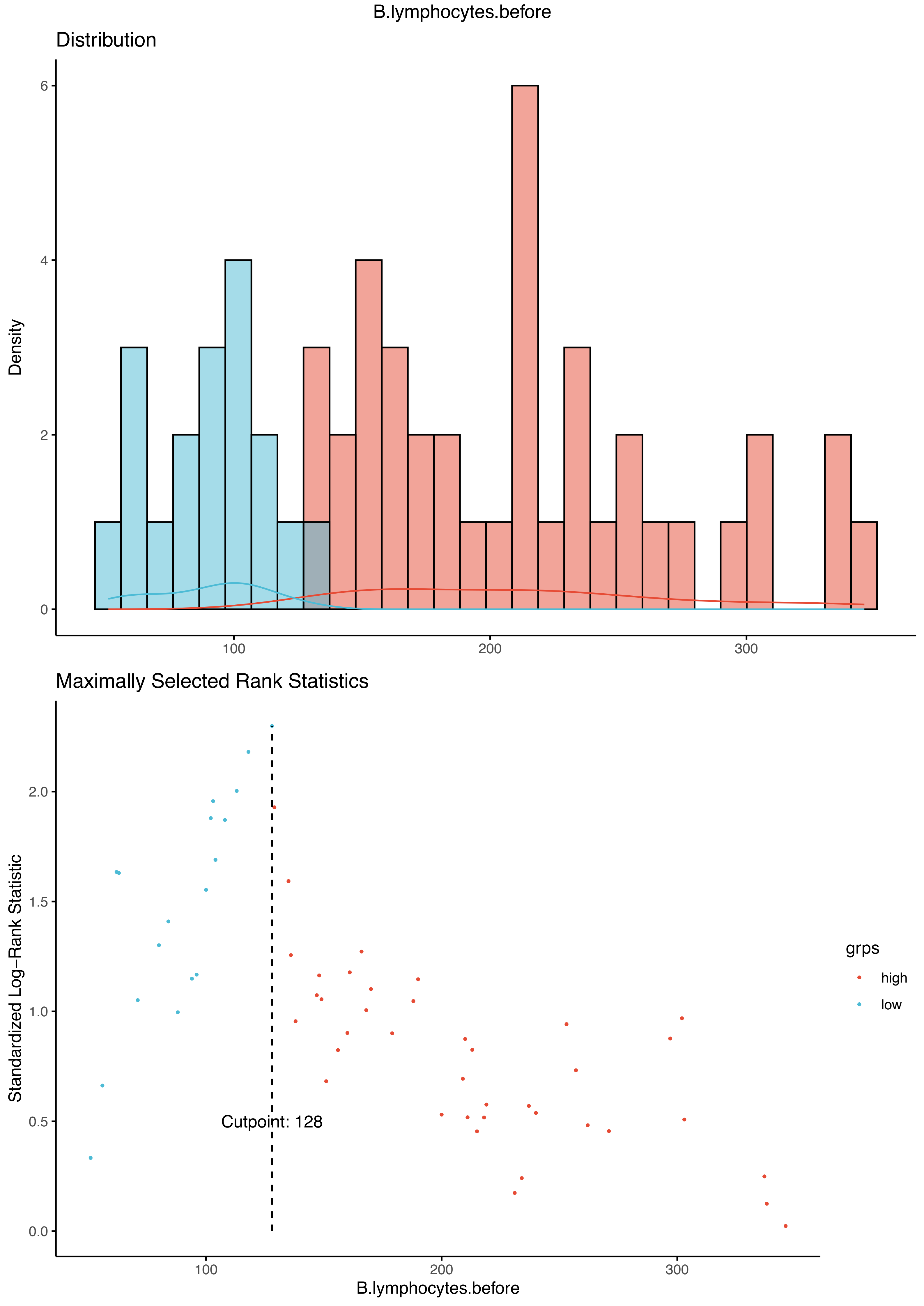
B.lymphocytes.before
Distribution
Density
0
2
4
6
100
200
300
Maximally Selected Rank Statistics
Standardized Log−Rank Statistic
0.0
0.5
1.0
1.5
2.0
Cutpoint: 128
100
200
300
B.lymphocytes.before
grps
high
low

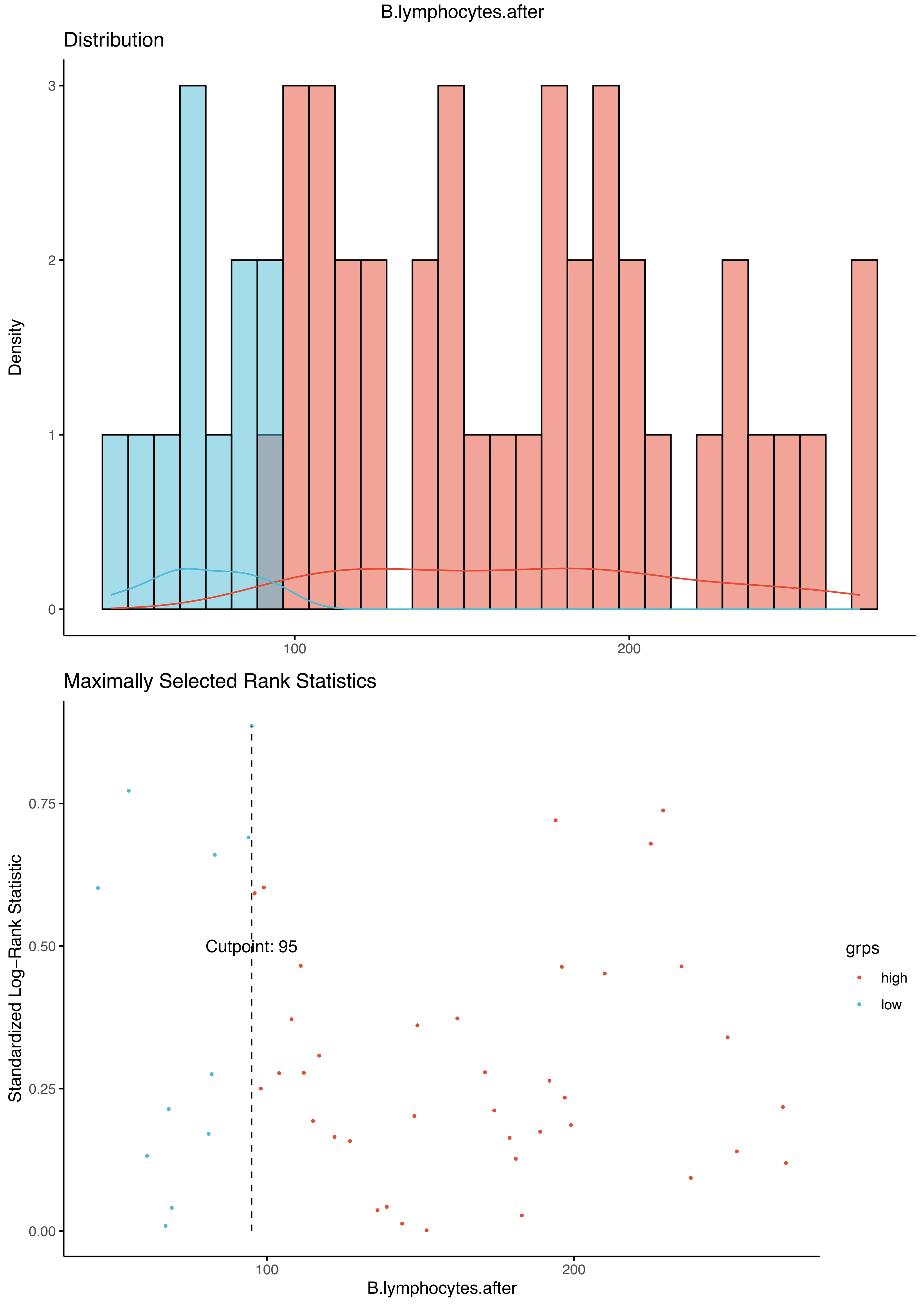
B.lymphocytes.after
Distribution
Density
3
2
1
0
100
200
Maximally Selected Rank Statistics
Standardized Log−Rank Statistic
0.75
0.50
0.25
0.00
Cutpoint: 95
grps
high
low
100
200
B.lymphocytes.after

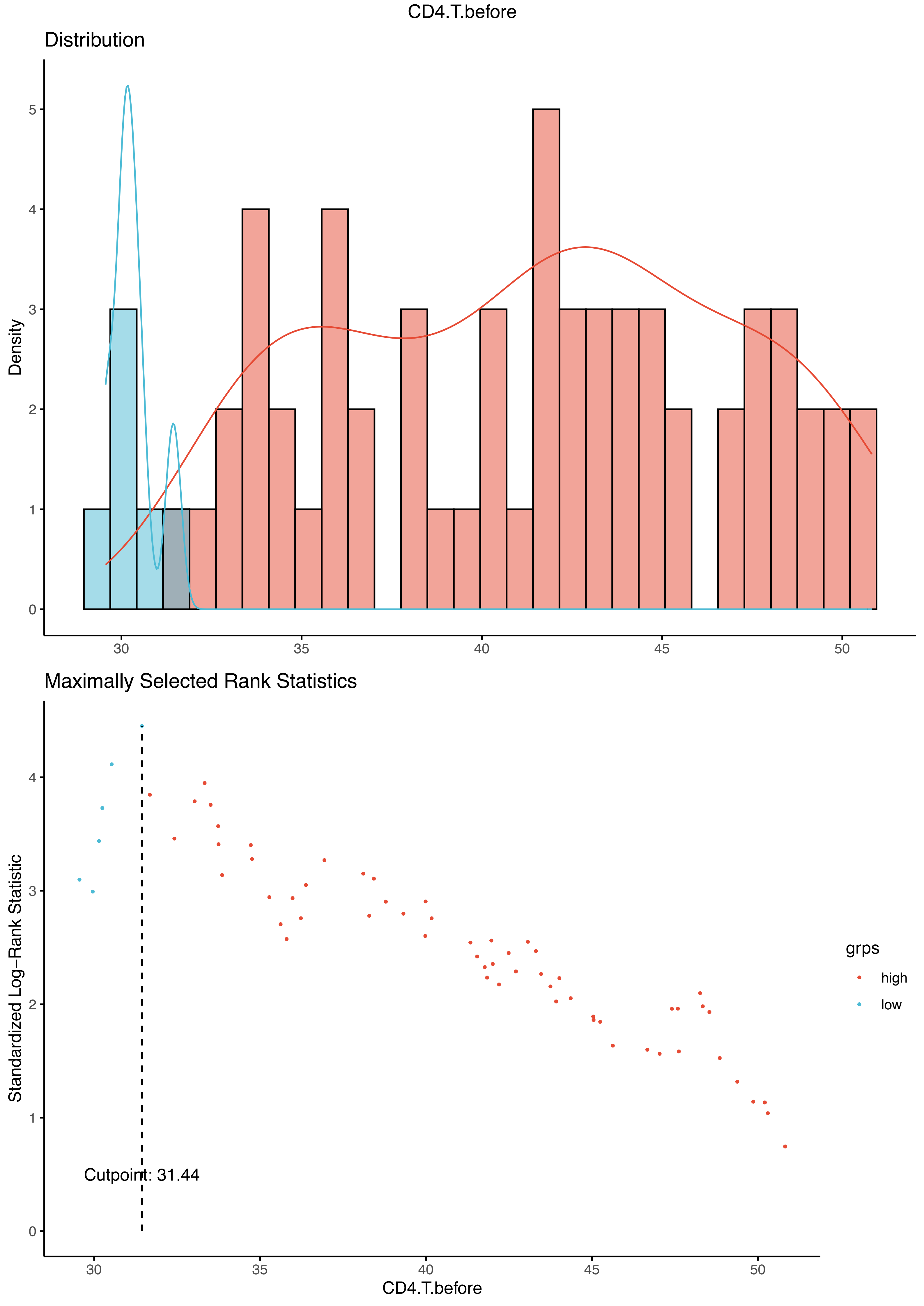

CD4.T.before
Distribution
Density
0
1
2
3
4
5
30
35
40
45
50
Maximally Selected Rank Statistics
Standardized Log−Rank Statistic
0
1
2
3
4
Cutpoint: 31.44
30
35
40
45
50
CD4.T.before
grps
high
low

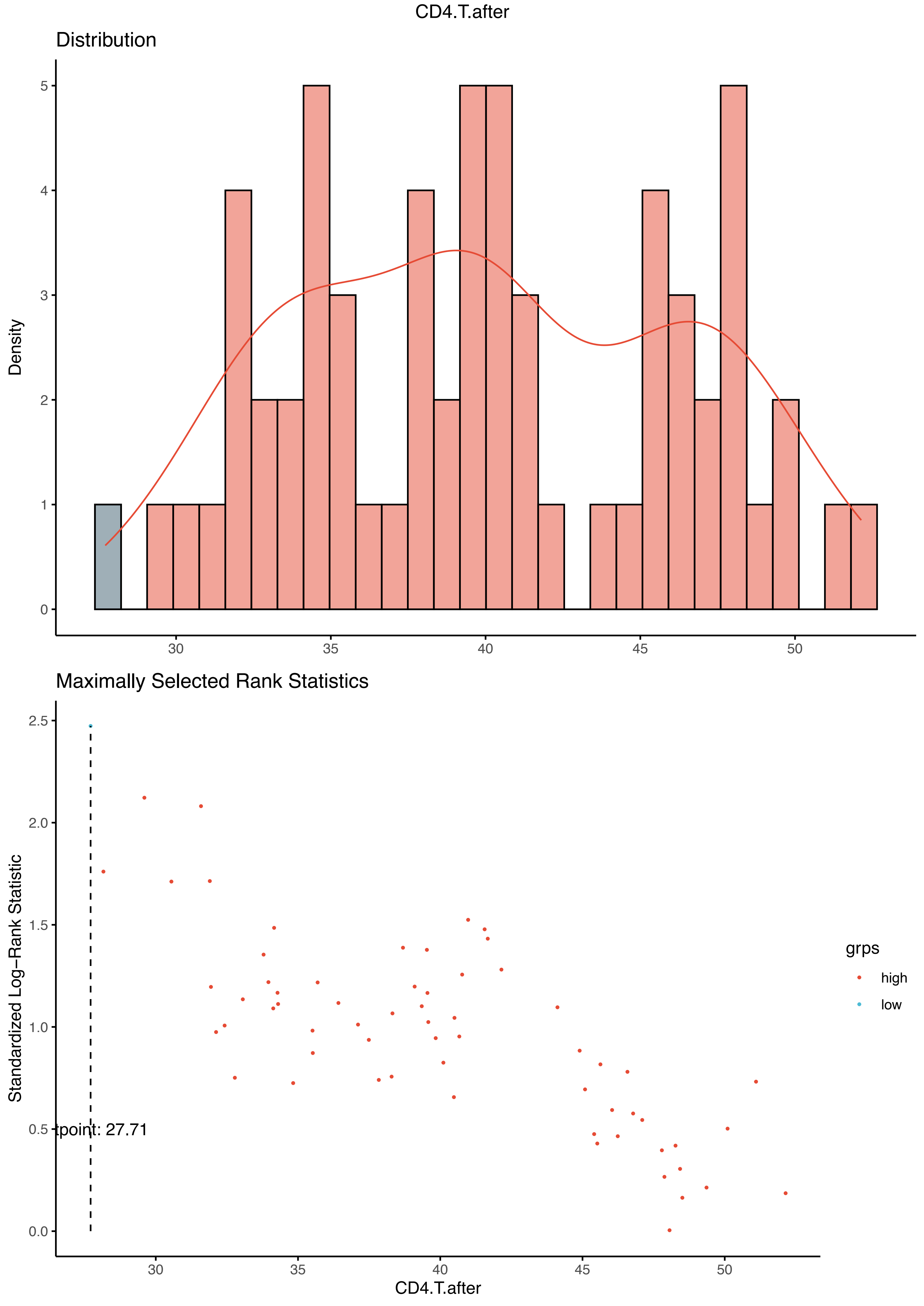
CD4.T.after
Distribution
Density
0
1
2
3
4
5
30
35
40
45
50
Maximally Selected Rank Statistics
Standardized Log−Rank Statistic
0.0
0.5
1.0
1.5
2.0
2.5
tpoint: 27.71
grps
high
low
CD4.T.after

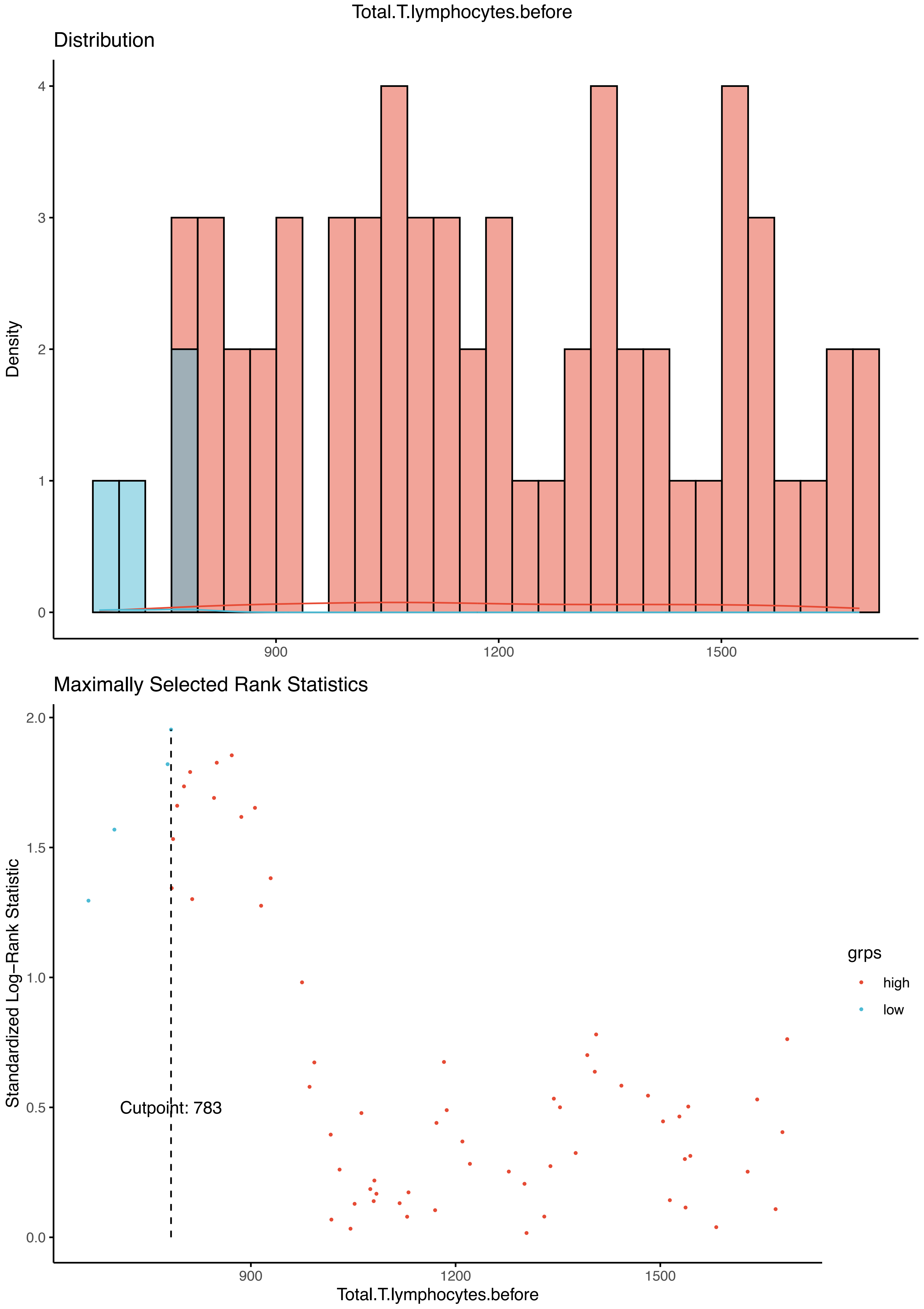
Total.T.lymphocytes.before
Distribution
Density
0
1
2
3
4
900
1200
1500
Maximally Selected Rank Statistics
Standardized Log−Rank Statistic
0.0
0.5
1.0
1.5
2.0
Cutpoint: 783
900
1200
1500
Total.T.lymphocytes.before
grps
high
low

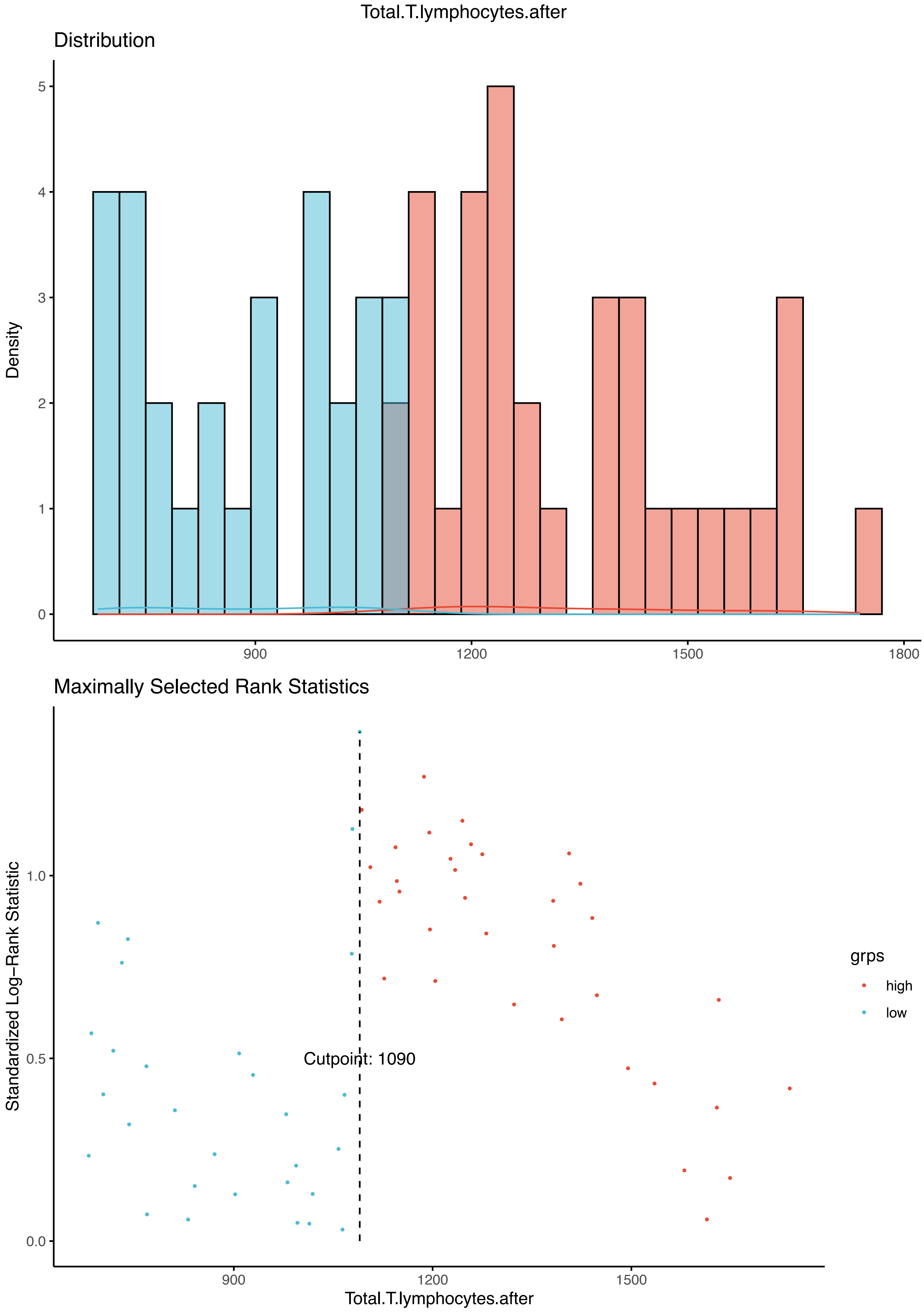

Total.T.lymphocytes.after
Distribution
Density
0
1
2
3
4
5
900
1200
1500
1800
Maximally Selected Rank Statistics
Standardized Log−Rank Statistic
0.0
0.5
1.0
Cutpoint: 1090
grps
high
low
900
1200
1500
Total.T.lymphocytes.after

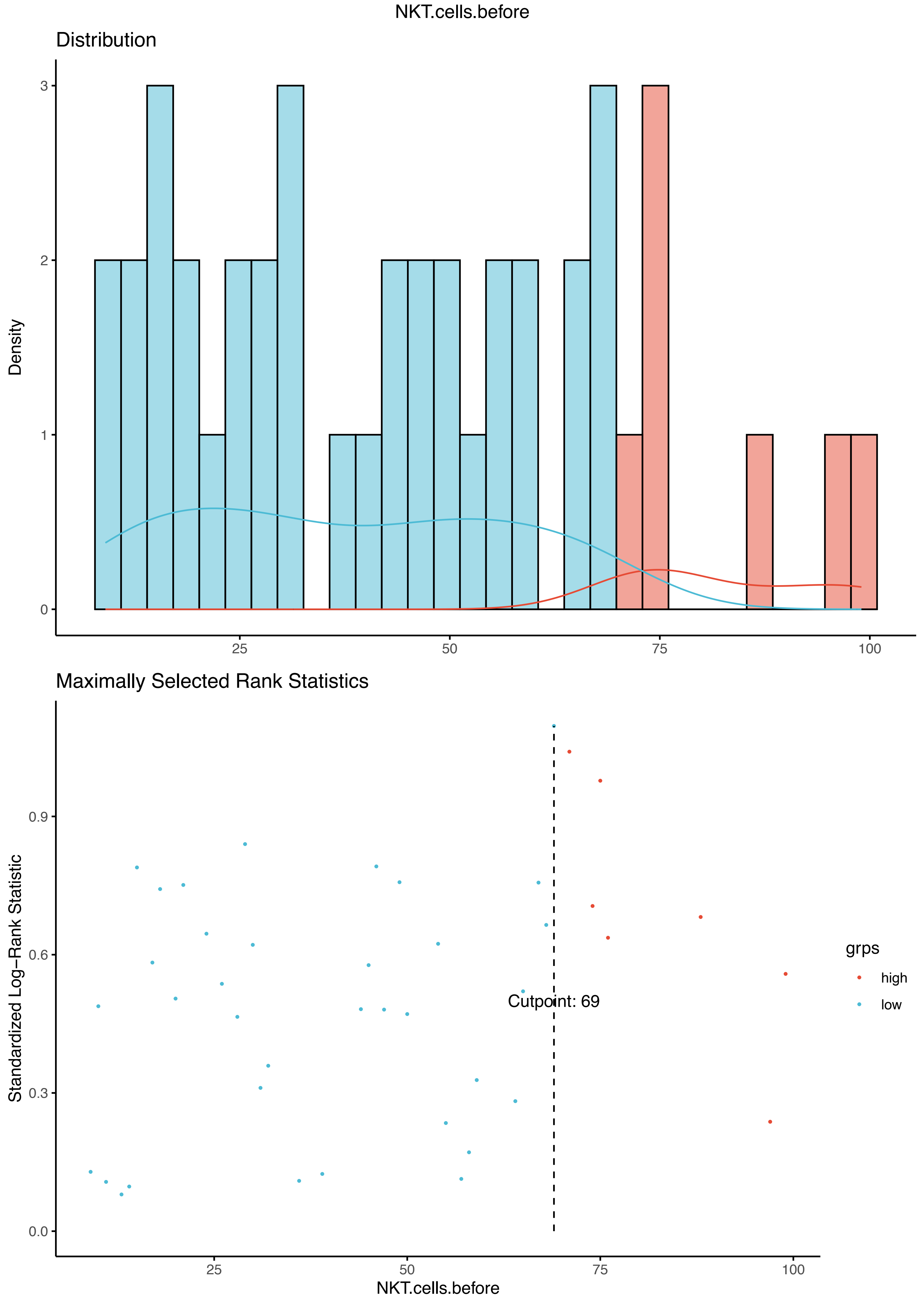
NKT.cells.before
Distribution
Density
0
1
2
3
25
50
75
100
Maximally Selected Rank Statistics
Standardized Log−Rank Statistic
0.0
0.3
0.6
0.9
Cutpoint: 69
grps
high
low
25
50
75
100
NKT.cells.before

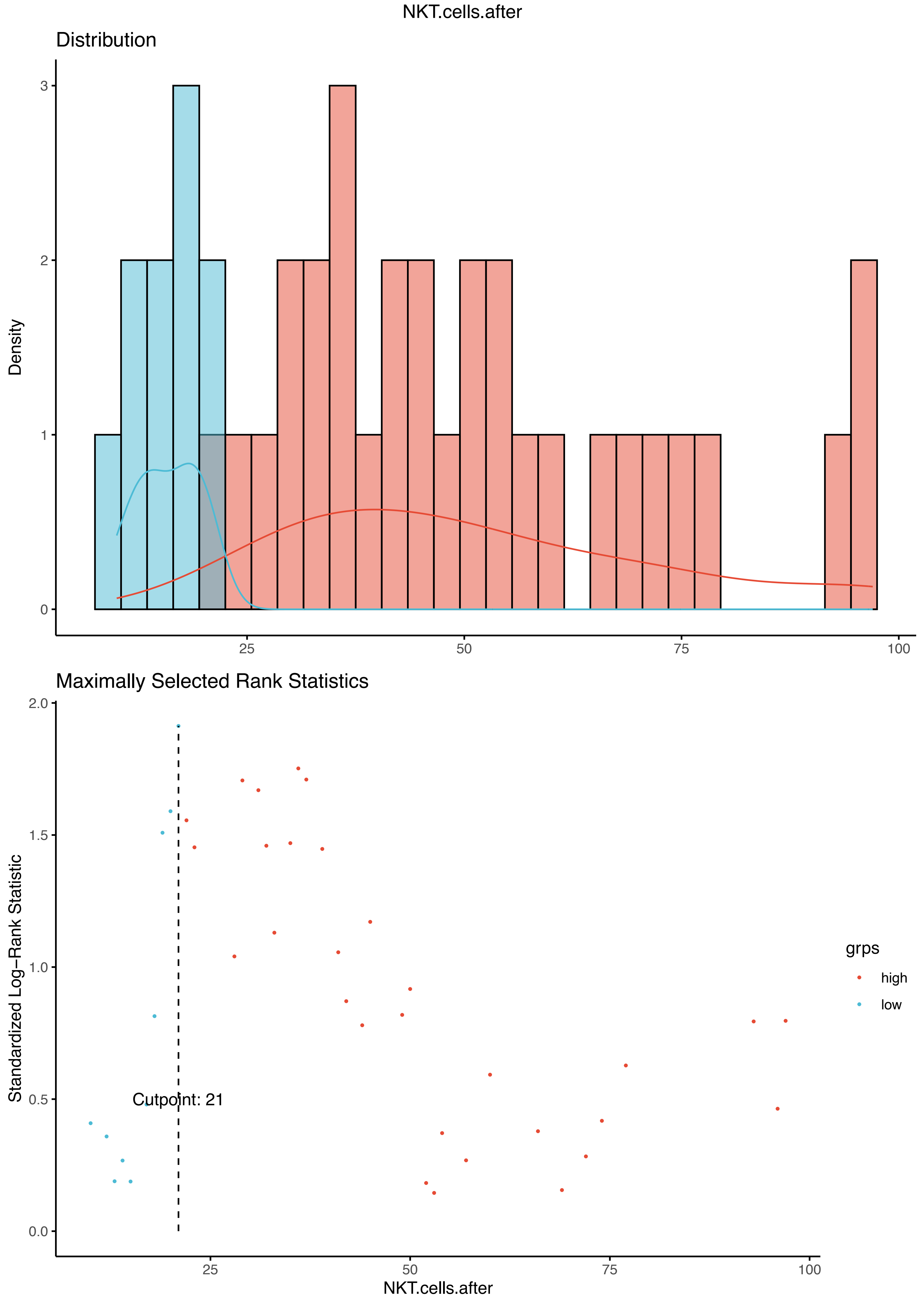
NKT.cells.after
Distribution
Density
3
2
1
0
25
50
75
100
Maximally Selected Rank Statistics
Standardized Log−Rank Statistic
2.0
1.5
1.0
0.5
0.0
Cutpoint: 21
25
50
75
100
NKT.cells.after
grps
high
low

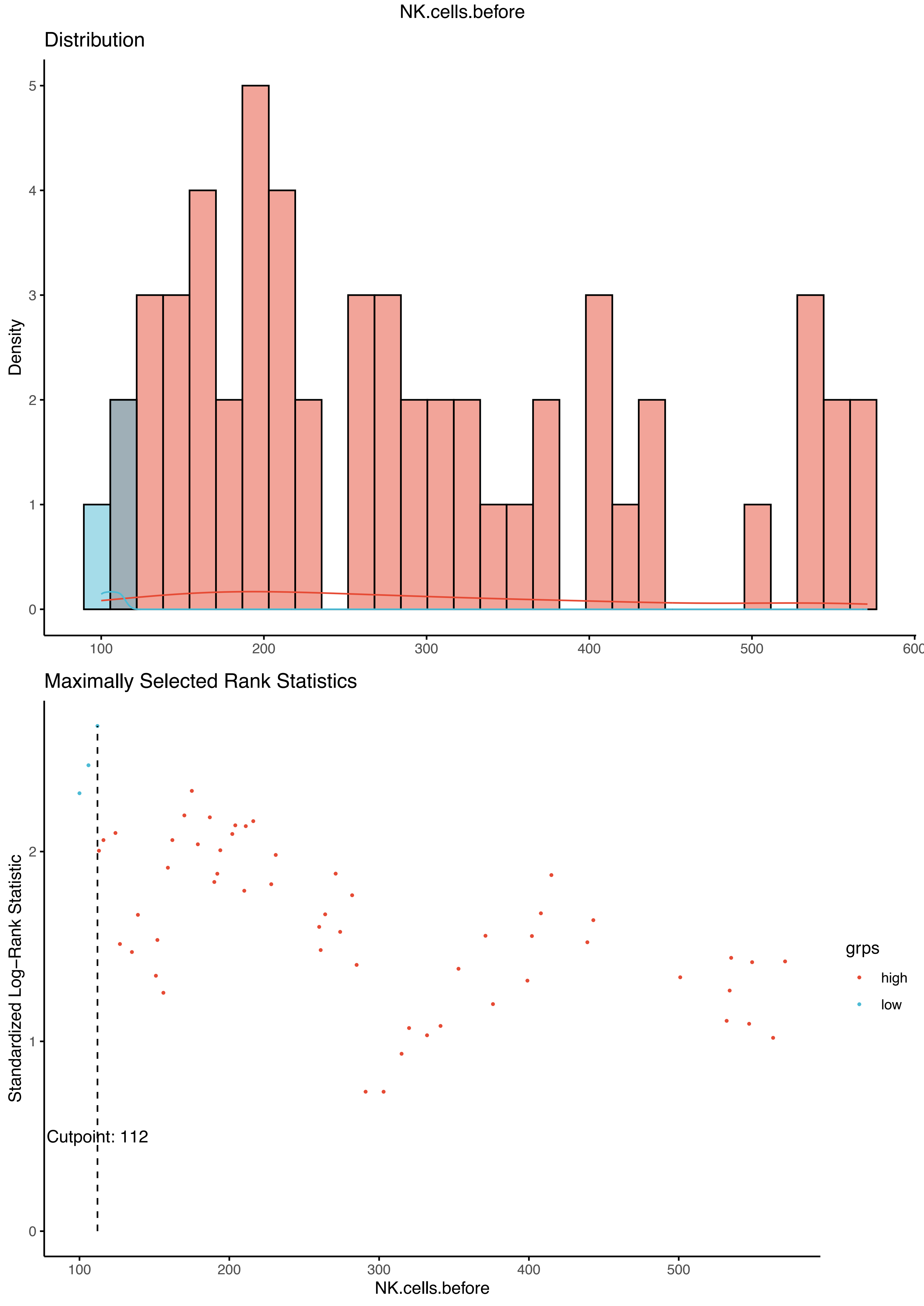

NK.cells.before
Distribution
Density
0
1
2
3
4
5
100
200
300
400
500
600
Maximally Selected Rank Statistics
Standardized Log−Rank Statistic
Cutpoint: 112
0
1
2
100
200
300
400
500
NK.cells.before
grps
high
low

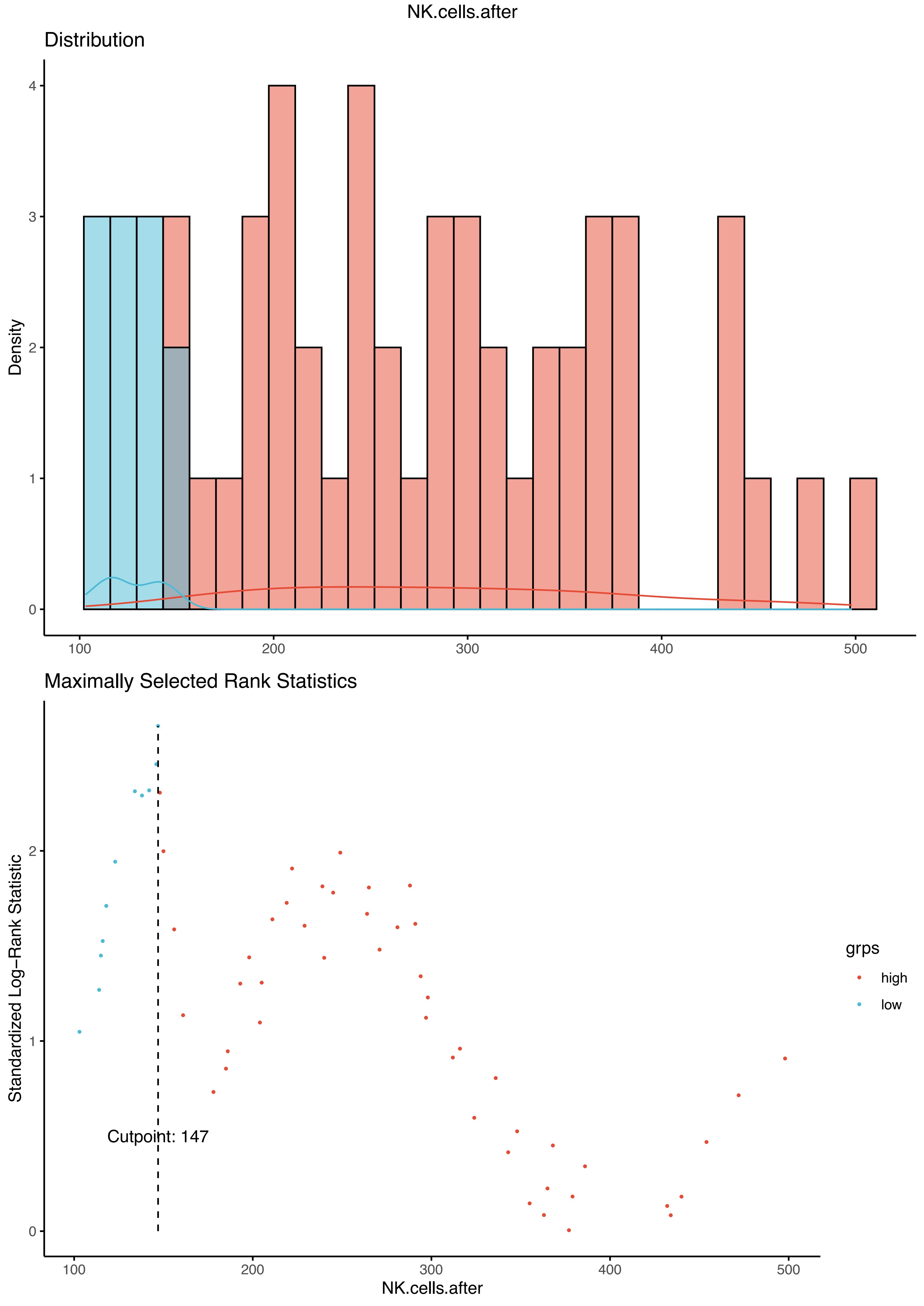

NK.cells.after
Distribution
Density
0
1
2
3
4
100
200
300
400
500
Maximally Selected Rank Statistics
Standardized Log−Rank Statistic
Cutpoint: 147
grps
high
low
NK.cells.after

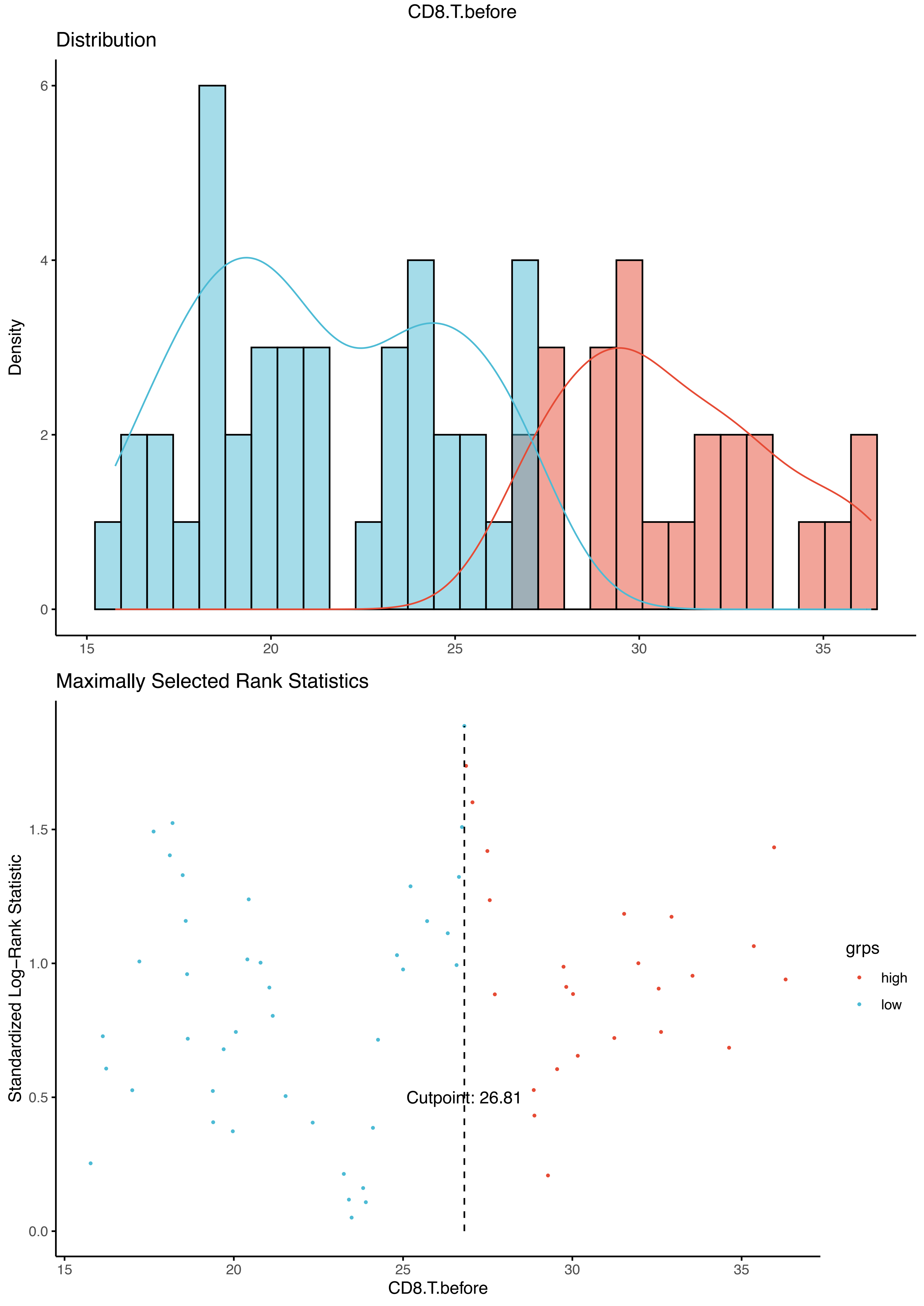
CD8.T.before
Distribution
Density
0
2
4
6
15
20
25
30
35
Maximally Selected Rank Statistics
Standardized Log−Rank Statistic
0.0
0.5
1.0
1.5
Cutpoint: 26.81
grps
high
low
15
20
25
30
35
CD8.T.before

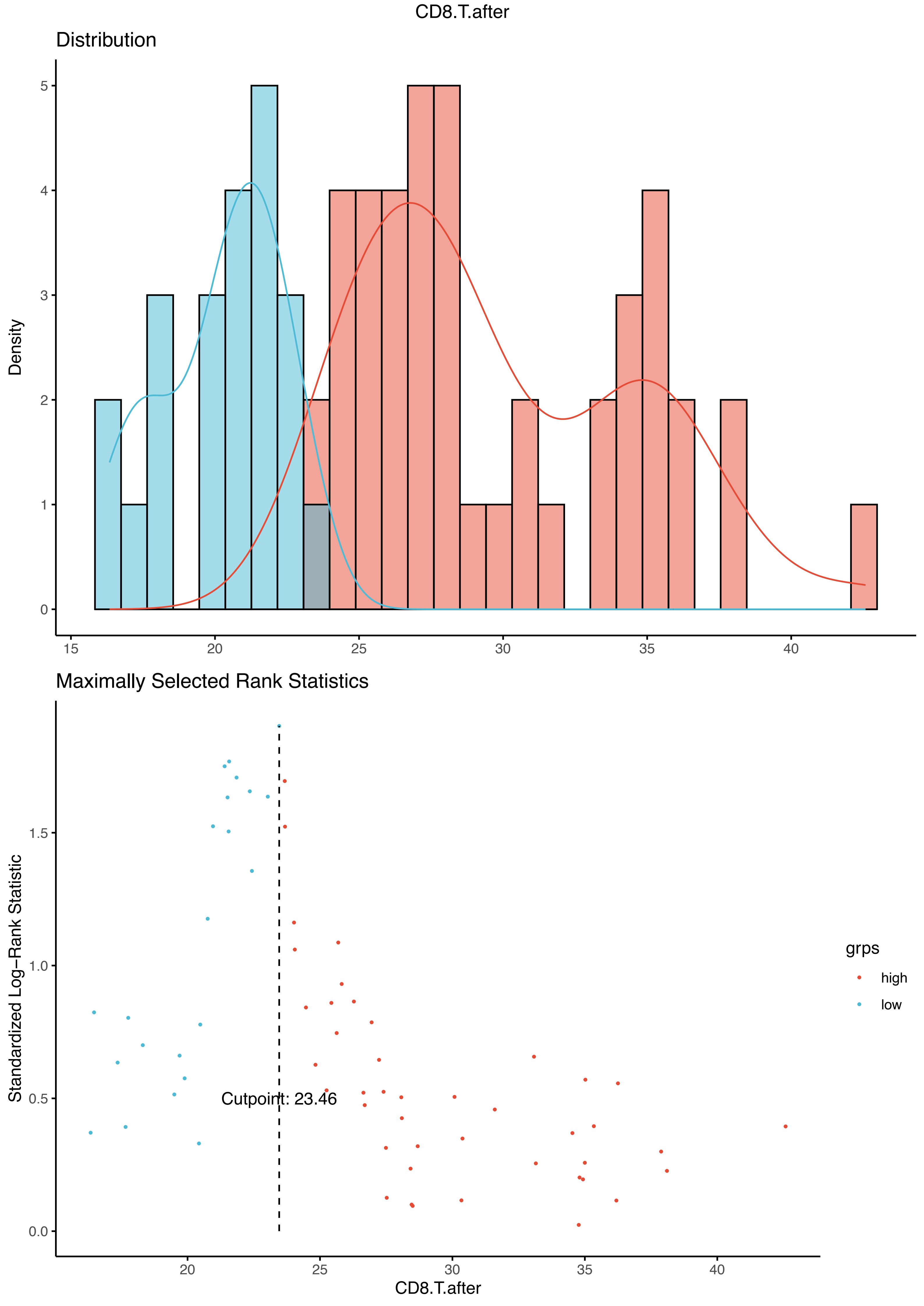
CD8.T.after
Distribution
Density
Maximally Selected Rank Statistics
Standardized Log−Rank Statistic
Cutpoint: 23.46
CD8.T.after
grps
high
low

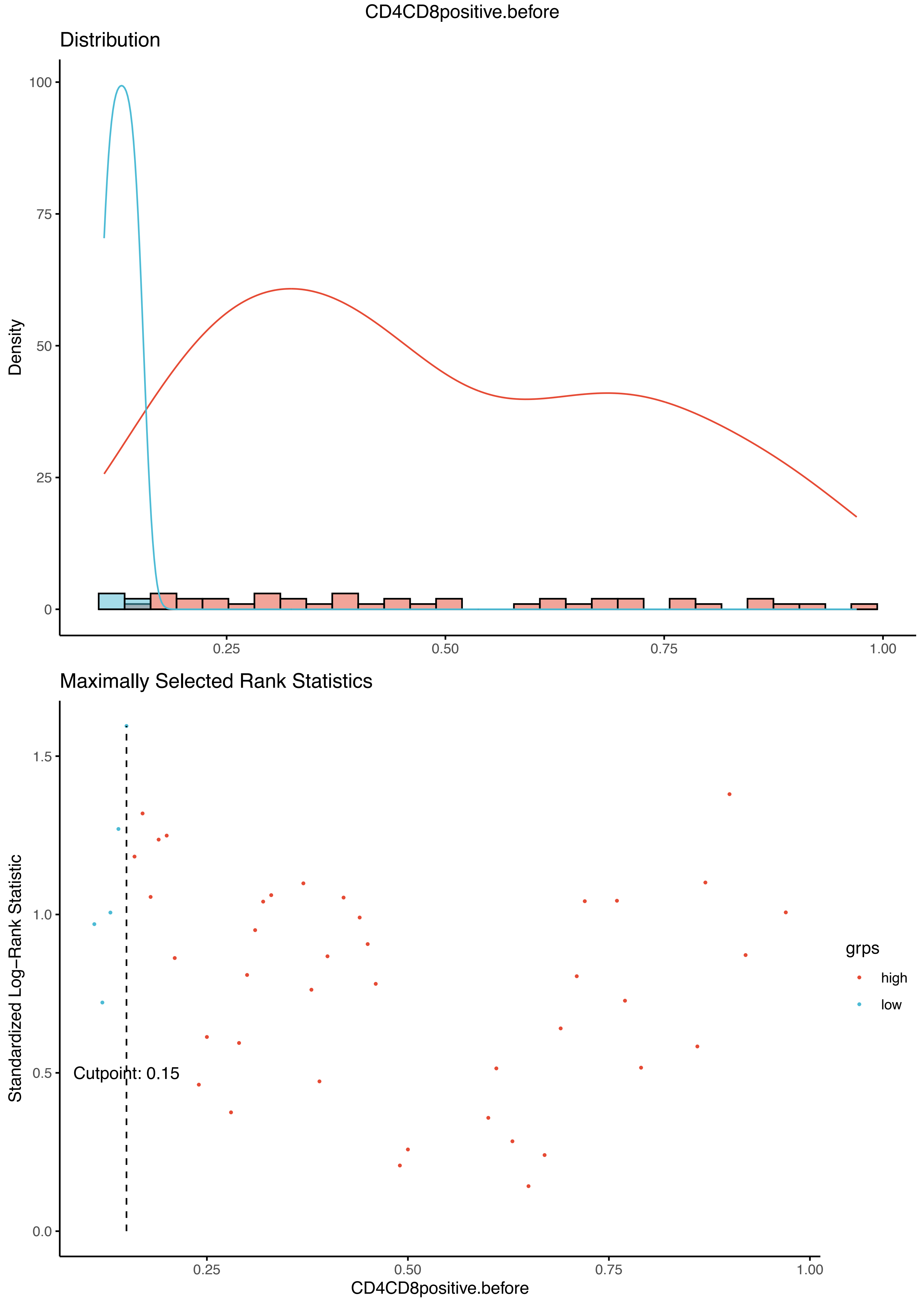
CD4CD8positive.before
Distribution
Density
100
75
50
25
0
0.25
0.50
0.75
1.00
Maximally Selected Rank Statistics
Standardized Log−Rank Statistic
1.5
1.0
0.5
0.0
Cutpoint: 0.15
0.25
0.50
0.75
1.00
CD4CD8positive.before
grps
high
low

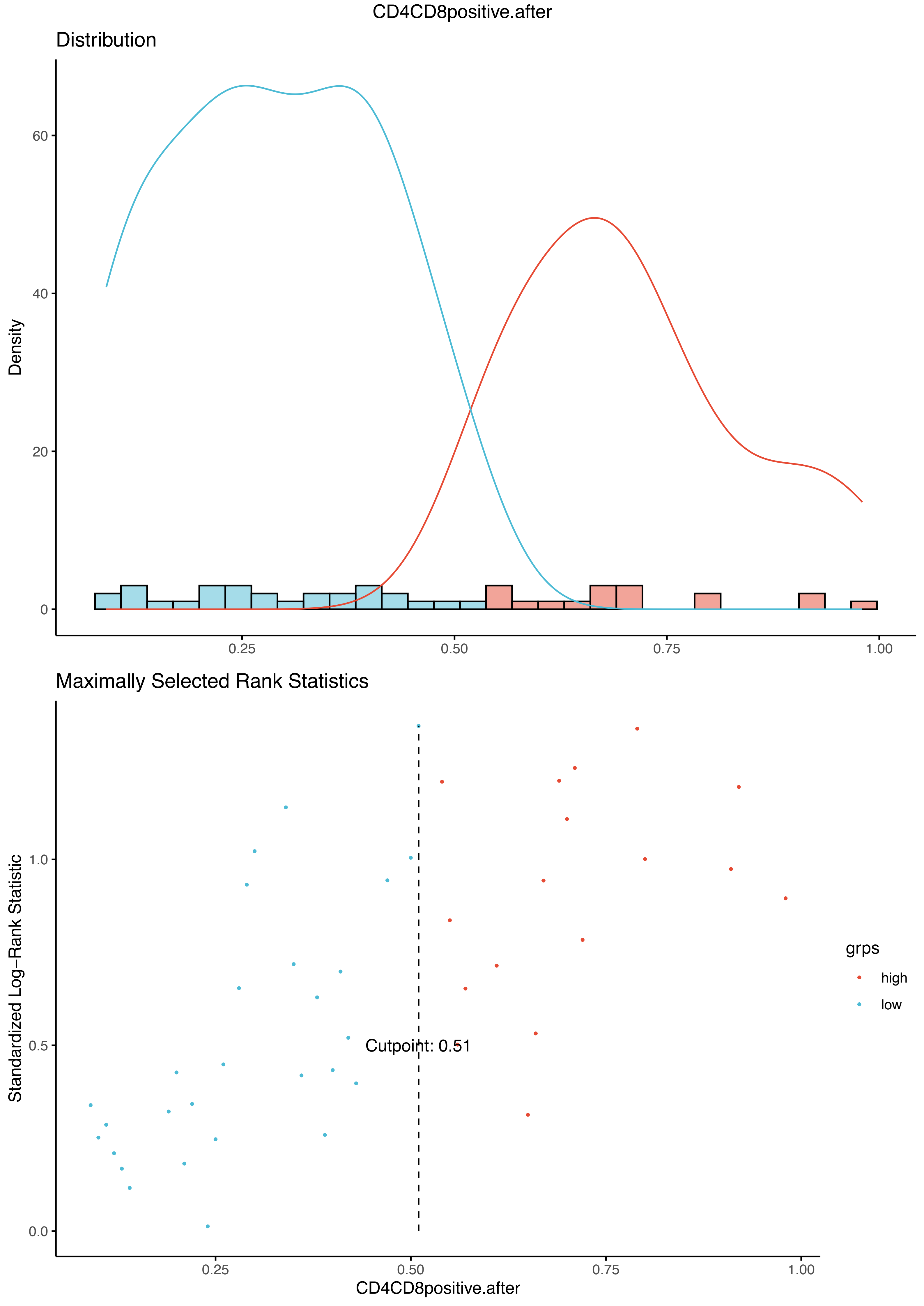

CD4CD8positive.after
Distribution
Density
60
40
20
0
0.25
0.50
0.75
1.00
Maximally Selected Rank Statistics
Standardized Log−Rank Statistic
1.0
0.5
0.0
Cutpoint: 0.51
grps
high
low
0.25
0.50
0.75
1.00
CD4CD8positive.after

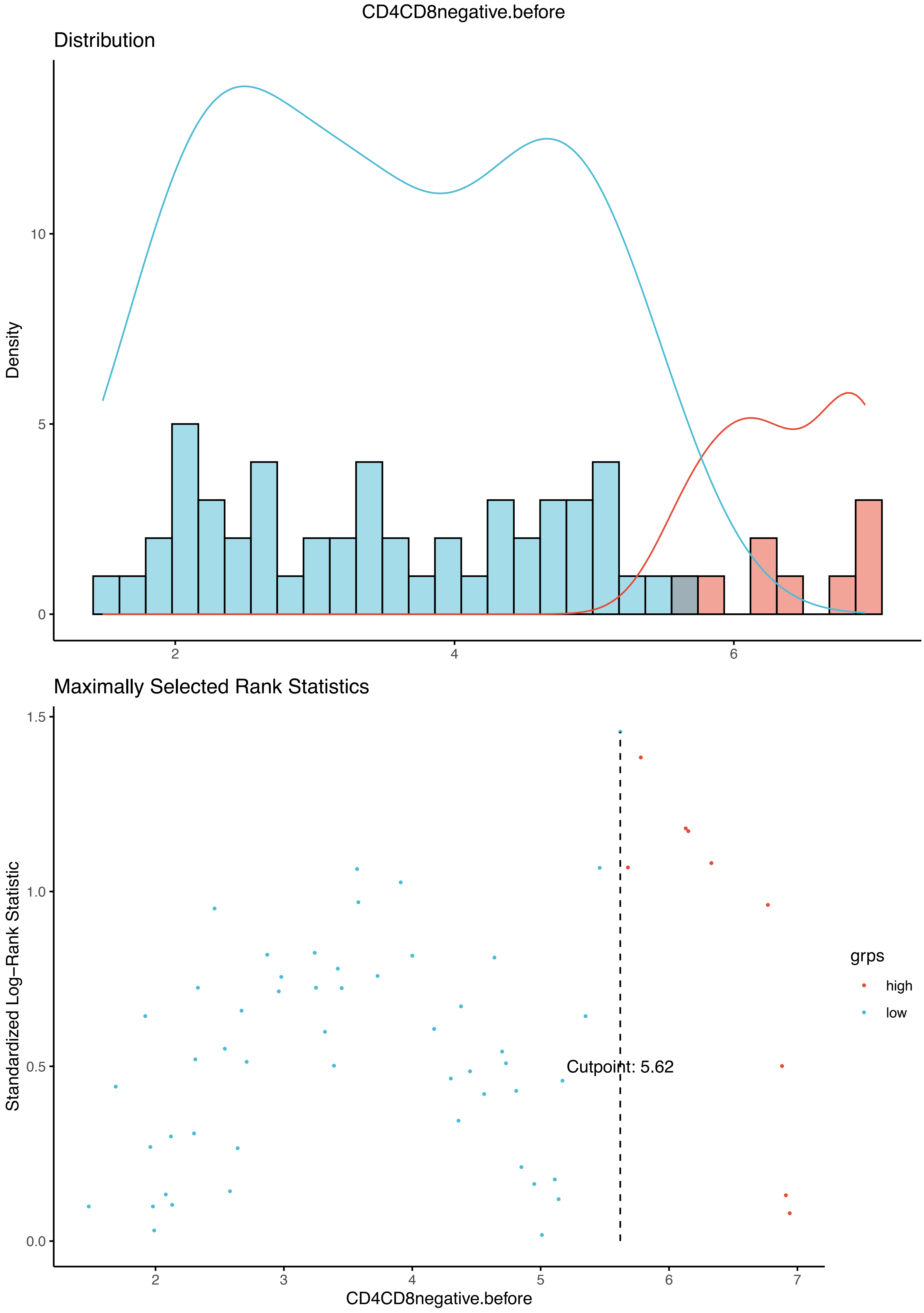
CD4CD8negative.before
Distribution
Density
10
5
0
2
4
6
Maximally Selected Rank Statistics
Standardized Log−Rank Statistic
1.5
1.0
0.5
0.0
Cutpoint: 5.62
grps
high
low
2
3
4
5
6
7
CD4CD8negative.before

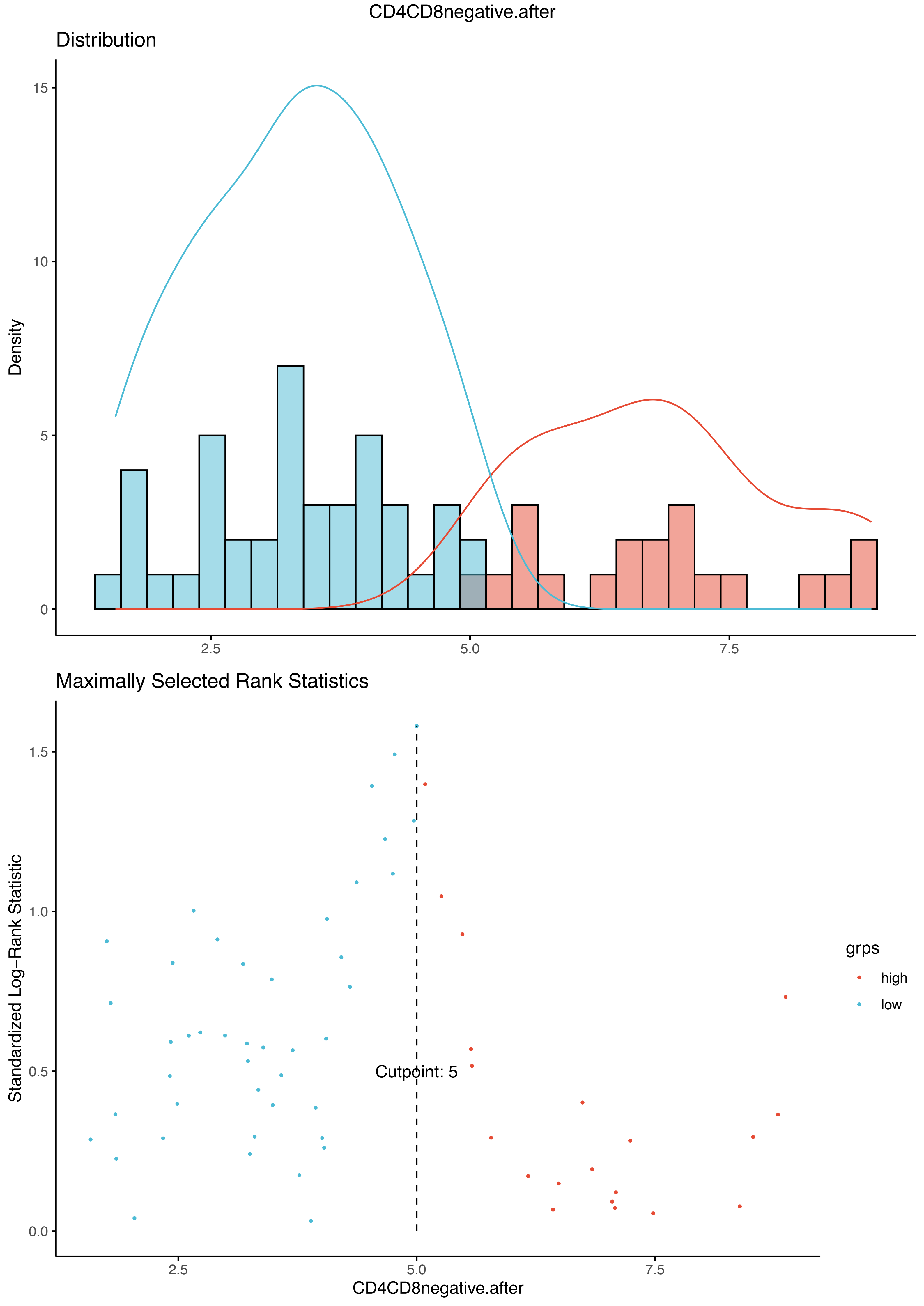
CD4CD8negative.after
Distribution
Density
15
10
5
0
2.5
5.0
7.5
Maximally Selected Rank Statistics
Standardized Log−Rank Statistic
1.5
1.0
0.5
0.0
Cutpoint: 5
grps
high
low
2.5
5.0
7.5
CD4CD8negative.after

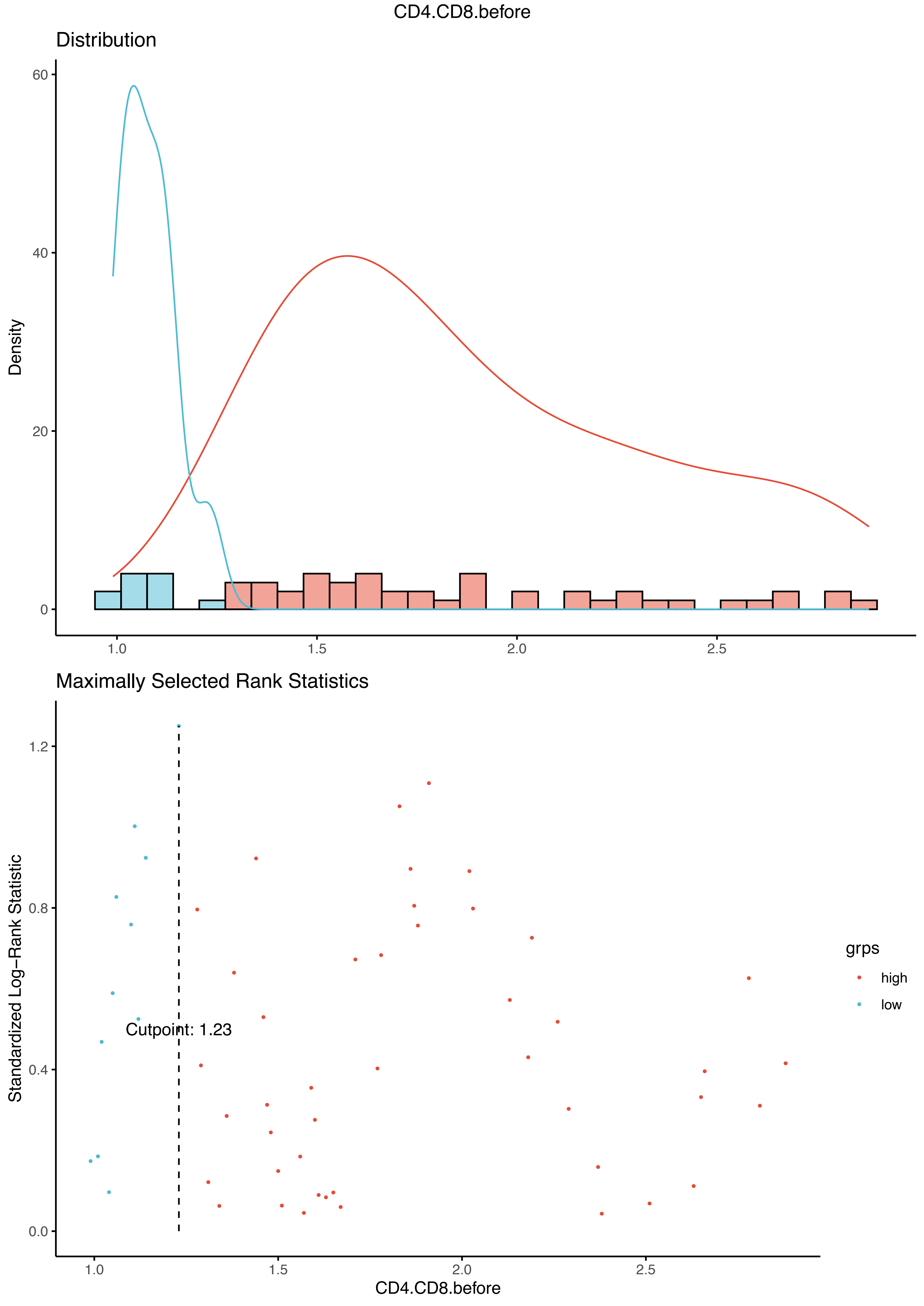

CD4.CD8.before
Distribution
Density
60
40
20
0
1.0
1.5
2.0
2.5
Maximally Selected Rank Statistics
Standardized Log−Rank Statistic
1.2
0.8
0.4
0.0
Cutpoint: 1.23
grps
high
low
CD4.CD8.before

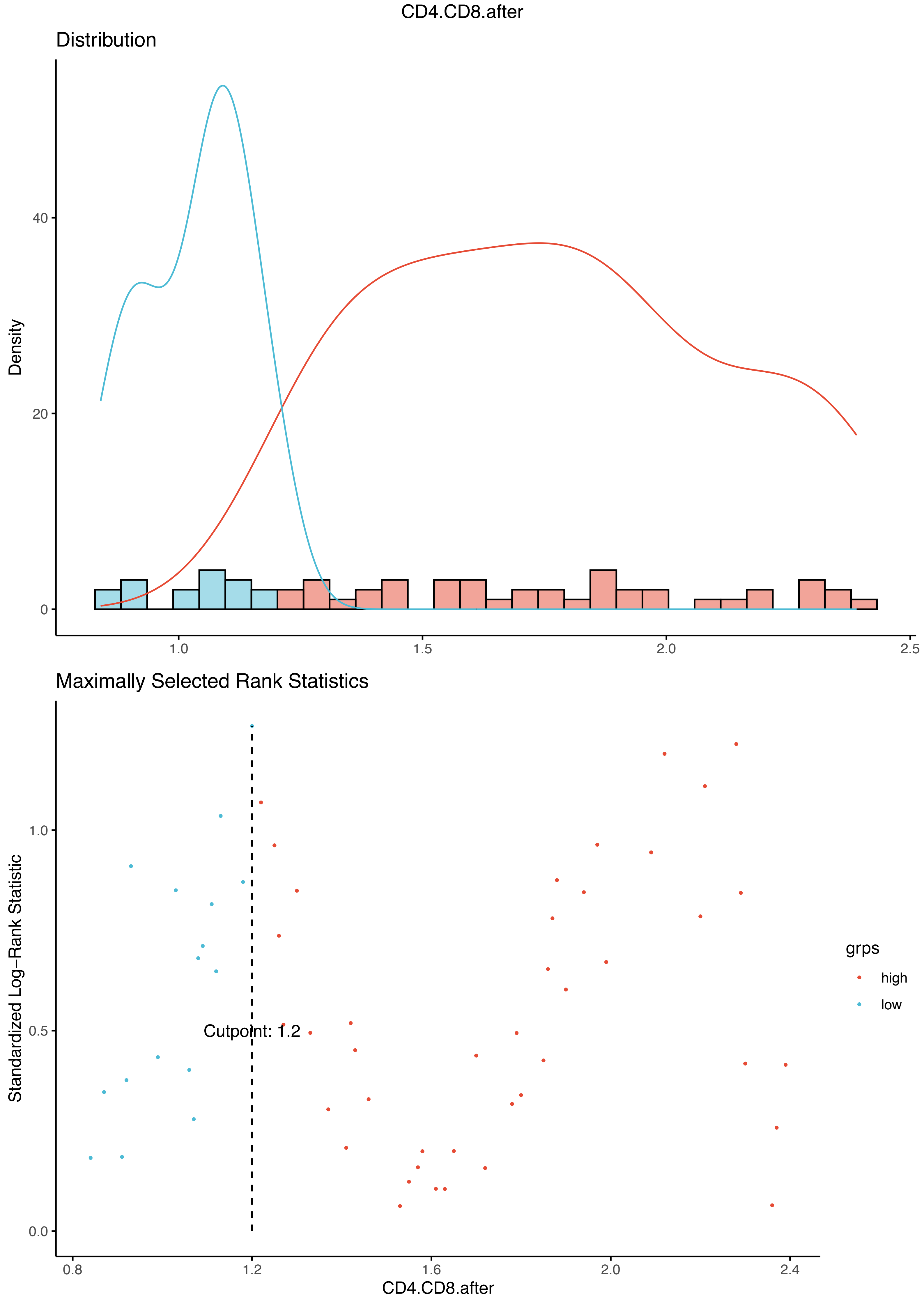
CD4.CD8.after
Distribution
Density
0
20
40
1.0
1.5
2.0
2.5
Maximally Selected Rank Statistics
Standardized Log−Rank Statistic
0.0
0.5
1.0
Cutpoint: 1.2
0.8
1.2
1.6
2.0
2.4
CD4.CD8.after
grps
high
low
