# Supplementary figures and images for: Peripheral CD4+ T cells correlate with response and survival in patients with advanced non-small cell lung cancer receiving chemo-immunotherapy
Source: Front Immunol. 2024 Apr 8;15:1364507. doi: 10.3389/fimmu.2024.1364507 (PMC11033411; doi:10.3389/fimmu.2024.1364507)

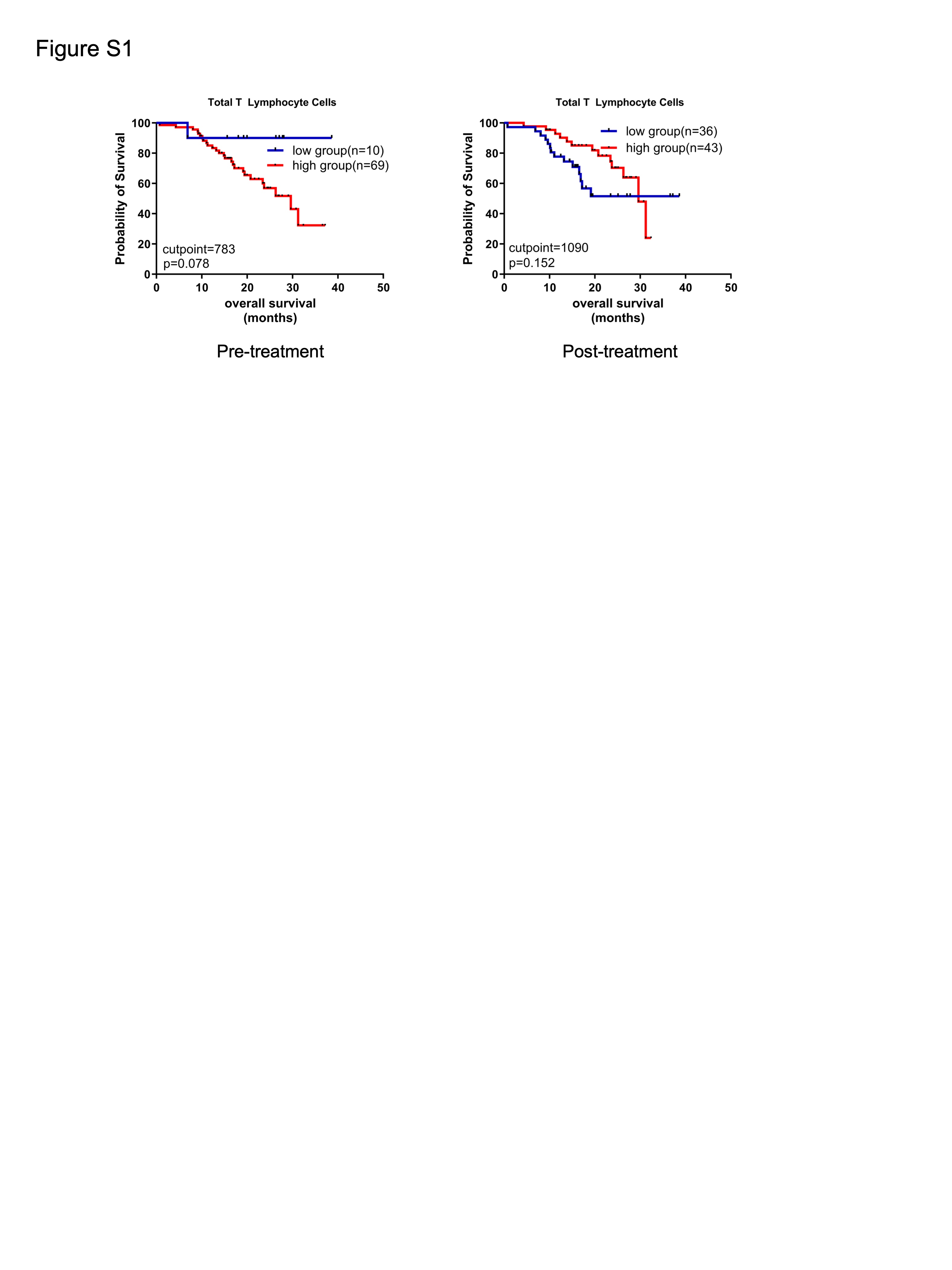

Supplement: Supplementary Figure S1 — Kaplan-Meier analysis on the overall survival. The Kaplan-Meier curves of OS of patients stratified by the optimal cutpoint of total T lymphocyte cells at pre- and post-treatment. [file Image_1.jpeg]

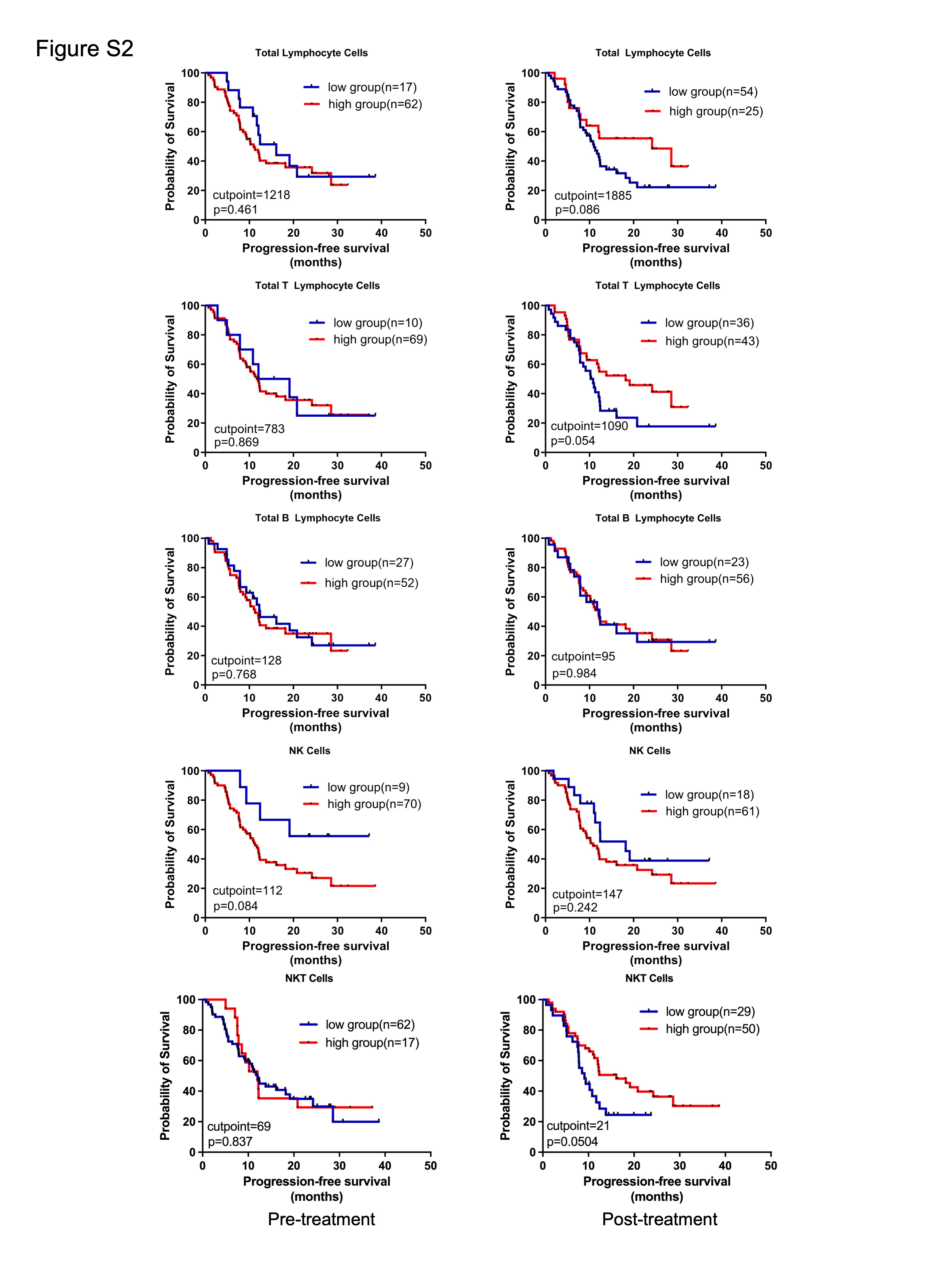

Supplement: Supplementary Figure S2 — Kaplan-Meier analysis on the progression-free survival. The Kaplan-Meier curves of PFS of patients stratified by the optimal cutpoints of OS at pre- and post-treatment. [file Image_2.jpeg]

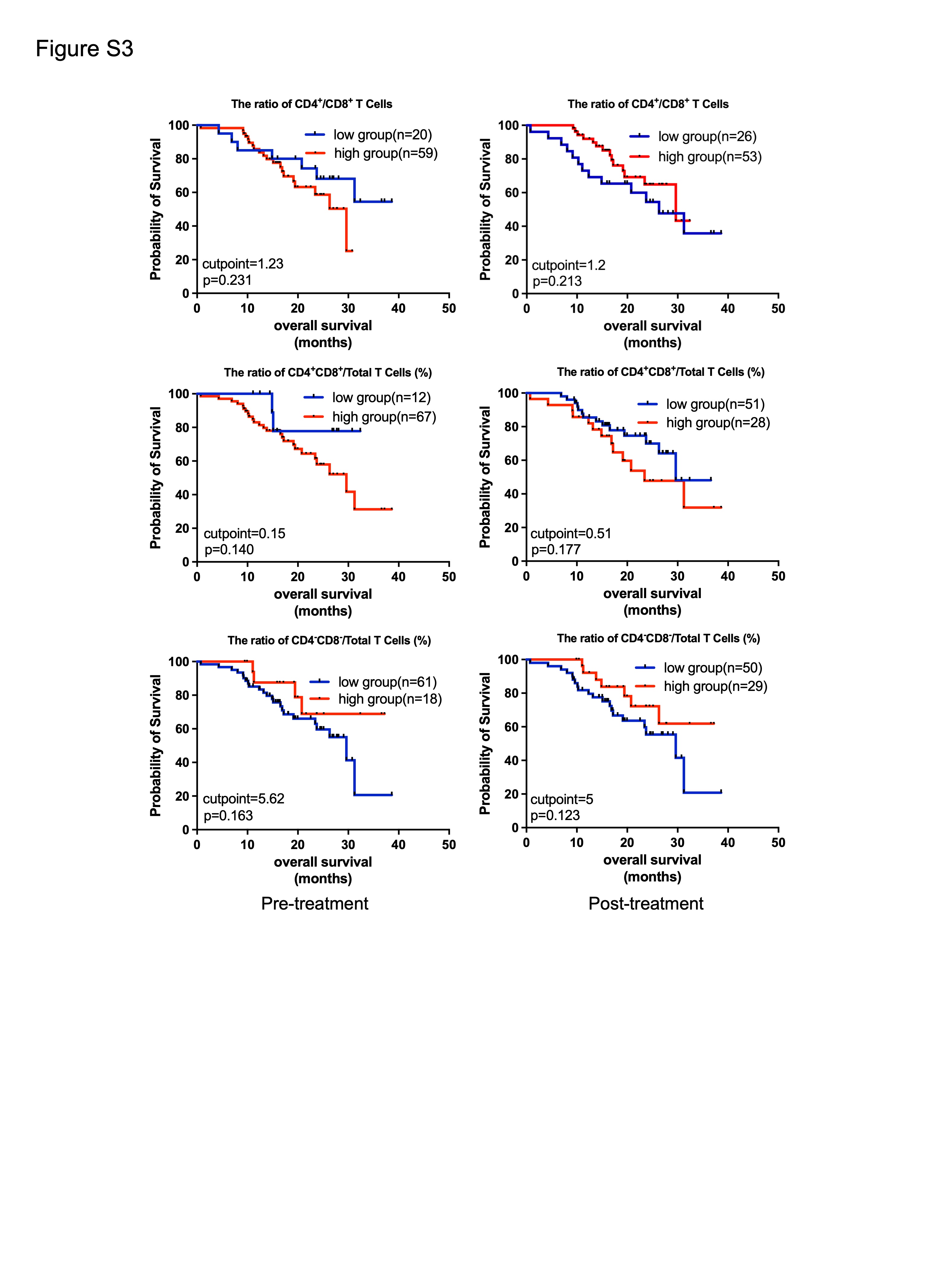

Supplement: Supplementary Figure S3 — Kaplan-Meier analysis on the overall survival. The Kaplan-Meier curves of OS of patients stratified by the optimal cutpoints of OS at pre- and post-treatment. [file Image_3.jpeg]

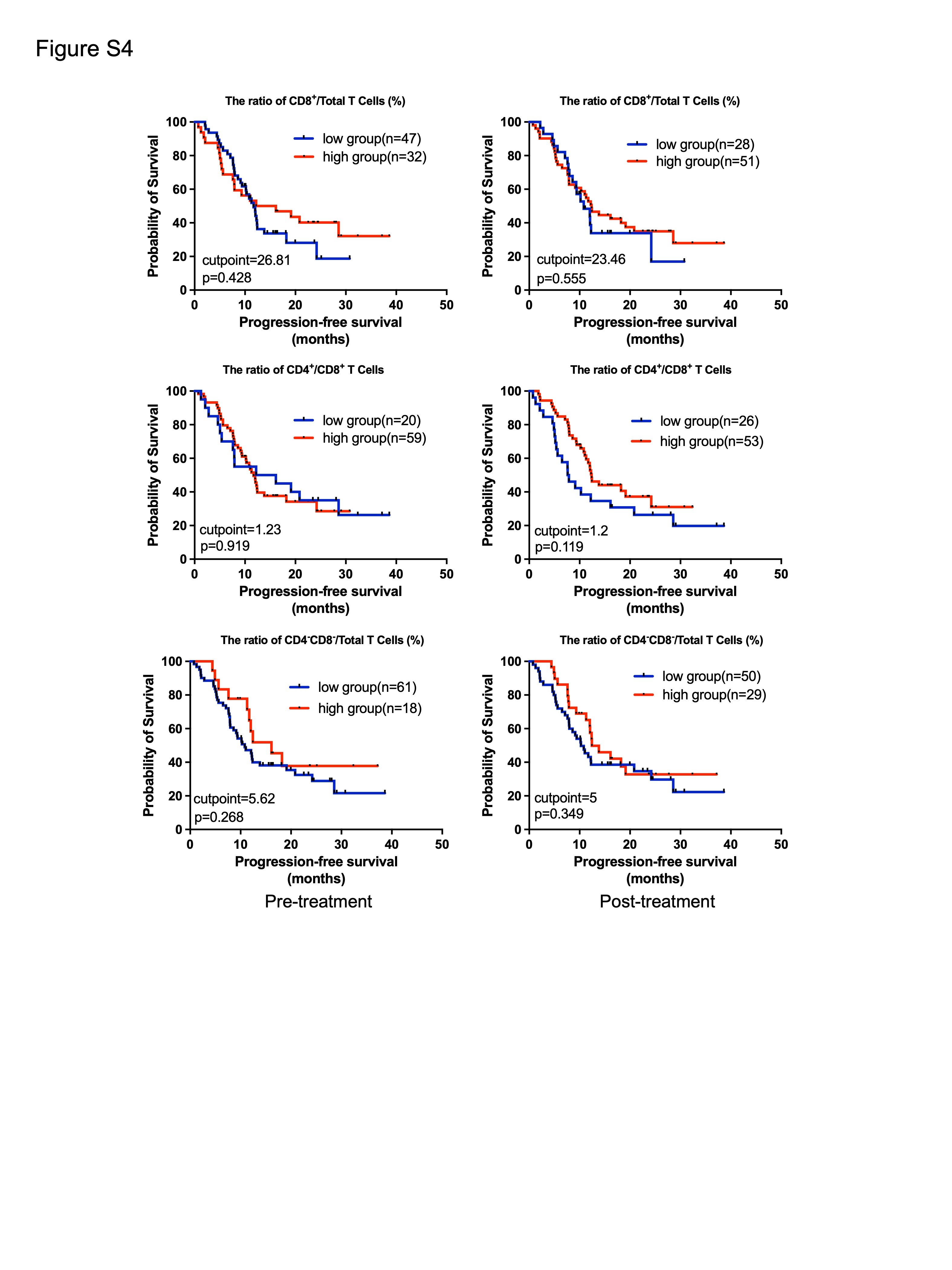

Supplement: Supplementary Figure S4 — Kaplan-Meier analysis on the progression-free survival. The Kaplan-Meier curves of PFS at pre- and post-treatment. The cutpoints for PFS were determined by the optimal cutpoints of OS. [file Image_4.jpeg]
