# Supplementary material for: Peripheral CD4+ T cells correlate with response and survival in patients with advanced non-small cell lung cancer receiving chemo-immunotherapy
Source: Front Immunol. 2024 Apr 8;15:1364507. doi: 10.3389/fimmu.2024.1364507 (PMC11033411; doi:10.3389/fimmu.2024.1364507)
Supplement: Supplementary file 6 [file Table_1.docx]

| **Table S1. Lists of antiboies used for immune phenotype analysis of PBMCs** | | | | |
| --- | --- | --- | --- | --- |
| **Antibody** | **Clone** | **Fluorochrome** | **Manufacturer** | **Reference** |
| CD3 | UCHT1 | FITC | QuantoBio | Z8610002 |
| CD4 | RPA-T4 | PC7 | QuantoBio | Z8610002 |
| CD8 | HIT8a | APC-Cy7 | QuantoBio | Z8610002 |
| CD16 | CB16 | PE | QuantoBio | Z8610002 |
| CD19 | HIB19 | APC | QuantoBio | Z8610002 |
| CD56 | MEM-188 | PE | QuantoBio | Z8610002 |
| CD45 | 2D1 | PerCP-Cy5.5 | QuantoBio | Z8610002 |
